# Exploring the chemical composition and processes of submicron aerosols in Delhi using aerosol chemical speciation monitor driven factor analysis

**DOI:** 10.1038/s41598-025-99245-9

**Published:** 2025-04-24

**Authors:** Upasana Panda, Supriya Dey, Amit Sharma, Aishwarya Singh, Ernesto Reyes-Villegas, Eoghan Darbyshire, Samara Carbone, Trupti Das, James Allan, Gordon McFiggans, R. Ravikrishna, Hugh Coe, Pengfei Liu, Sachin S. Gunthe

**Affiliations:** 1 EE Division, Department of Civil Engineering, Indian Institute of Technology Madras, Chennai, India; 2 Department of Environment and Sustainability, CSIR-Institute of Minerals and Materials Technology, Bhubaneswar, India; 3 Centre for Atmospheric and Climate Sciences, Indian Institute of Technology Madras, Chennai, India; 4 Department of Civil and Infrastructure Engineering, Indian Institute of Technology Jodhpur, Karwar, Jodhpur, India; 5 Department of Earth and Environmental Sciences, School of Natural Sciences, University of Manchester, Manchester, UK; 6 Escuela de Ingeniería y Ciencias, Tecnologico de Monterrey, Av. General Ramon Corona 2514, Nuevo México, 45138 Zapopan, Jalisco Mexico; 7 The Conflict and Environment Observatory, Hebden Bridge, West Yorkshire UK; 8 Institute of Agrarian Sciences, Federal University of Uberlândia, Uberlândia, MG Brazil; 9 National Centre for Atmospheric Science, University of Manchester, Manchester, UK; 10 Department of Chemical Engineering, Indian Institute of Technology Madras, Chennai, India; 11 School of Earth and Atmospheric Sciences, Georgia Institute of Technology, Atlanta, GA USA; 12 Present Address: Kalinga Institute of Industrial Technology-Deemed to be University, Bhubaneswar, India

**Keywords:** Tropical India, NR-PM_1_, Organic aerosol, ACSM, Source apportionment, PMF, Atmospheric science, Climate change

## Abstract

Wintertime non-refractory submicron particulate matter (NR-PM_1_) species were measured in Delhi with an Aerodyne Aerosol Chemical Speciation Monitor (ACSM) during February–March 2018. The average NR-PM_1_ mass concentration throughout the study was 58.0 ± 42.6 µg m^−3^, where the contribution of organic aerosol (OA) was 69% of the total NR-PM_1_. In Delhi, chloride (10%) was the main inorganic contributor, followed by ammonium (8%), sulfate (7%), and nitrate (6%), contrasting with the prevalence of sulfate in most urban environments. Source apportionment analysis of the OA identified five major factors, including three primary contributors: hydrocarbon-like OA (HOA), biomass burning OA (BBOA), cooking-related OA (COA) and two secondary contributors: oxygenated primary OA (OPOA), and more-oxidized oxygenated OA (MO-OOA). A 19% rise in OPOA concentration was observed during high chloride episodes, suggesting the potential role of chloride in the atmospheric chemical transformation of OA. Traffic emissions significantly contribute to ambient OA, accounting for at least 41% of the total OA mass. Furthermore, the OA exhibited low oxidation levels regardless of its source. The *f*_44_:*f*_43_ analysis revealed slower atmospheric oxidization of OA compared to other urban locations worldwide. Further investigations, including chamber experiments tailored to the Delhi atmosphere, are necessary to elucidate the atmospheric oxidants and the genesis of secondary OA alongside primary emissions.

## Introduction

Air pollution is transboundary and has become a serious threat to human beings over the last decades, particularly in urban environments^1,2^.The upshot of particulate matter (PM) as a pollutant in climate change is one of the most significant sources of uncertainty for climate prediction due to its complex role in atmospheric chemistry. In particular, the growing evidence on the effects of atmospheric submicron particulate matter with an aerodynamic diameter less than 1 µm (PM_1_), covers a wide range of topics from human health to climate change, making it one of the widely recognized air pollutants^3^.

Organic aerosols (OA) are the most complex composition of ambient PM_1_, and they contribute a significant fraction (around 50–85%) of total PM_1_ mass^4^. It can be emitted directly into the atmosphere, known as primary OA (POA), or chemically transformed as secondary products by several atmospheric phenomena through various multiphase oxidation reactions, known as secondary OA (SOA)^5^. In recent years, factor analysis of organic mass spectra has been extensively used to characterize OA sources precisely for laboratory and field measurements^6–11^.

Delhi, recognized as the world’s second most populous city, has garnered significant international attention and sparked fervent public debate due to escalating air pollution levels, particularly with the advent of winter, as indicated by the recent World Health Organization (WHO) estimates (2018)^12^. Over the past few decades, particulate pollution has emerged as a critical environmental issue in Delhi, with concentrations consistently exceeding legal threshold^13–16^. The city endures serious air quality-related challenges, including reduced visibility, extreme weather conditions, and elevated air toxicity, which collectively exert deleterious effects on human health and local ecosystems^17,18^. The highest concentrations of particulate matter (PM) are typically observed during winter months (December–February), coinciding with frequent severe pollution episodes. These pollution episodes are exacerbated by stable meteorological conditions that trap pollutants near the ground^19,20^. Research over this region predominantly focuses on PM_10_ and PM_2.5_ characterization, while less attention has been directed towards the measurement and analysis of submicron particles. Although national standards have been established for PM_10_ and PM_2.5_, there are currently no defined threshold limits or permissible standards for ambient PM_1_ levels set by any environmental protection agency, including the Central Pollution Control Board (CPCB) of India.

While many studies have been conducted characterizing various PM characteristics over Delhi^21–27^, few studies have paid detailed attention on characterizing PM_1_ mass concentrations, attributing various industries and surrounding thermal power plants^19,28–32^ as sources of primary emissions. Most of these studies focused on offline chemical characterization limited to ionic and elemental analyses, resulting in understanding, which is further limited due to low time resolution. Such studies could not explain the underlying mechanisms leading to rapid evolution of aerosols and accurate emission sources, which requires online and real-time information on high time resolution. Filter measurements may additionally suffer from handling errors during sampling and loss to semi-volatile particles by evaporation^33^. A detailed understanding of the regional characteristics of PM_1_ in Delhi, therefore, has regional and global importance towards designing prevention and control of megacity air pollution strategies for assessing human exposure to submicron particles and health risks. Air pollution control strategies require a thorough scientific understanding of the processes responsible for airborne particle formation and subsequent atmospheric transformation and ageing. Gani et al.^19^, during 2017–2018, carried out the first continuous online measurement of NR-PM_1_ reporting components for six consecutive seasons using ACSM in Delhi. The source apportionment of NR-PM_1_ using positive matrix factorization (PMF) analysis revealed that primary organic aerosol (POA), including HOA and BBOA, dominates during severe pollution episodes in New Delhi, while oxygenated organic aerosol (OOA), linked to secondary organic aerosol (SOA) and secondary inorganic species, contributes more to seasonal averages^32,34^. Cash et al.^10^ measured PM_1_ composition using high-resolution mass spectrometry (HR-AMS) and the PMF analysis identified two factors for crop residue and solid fuel burning, and two traffic-related hydrocarbon-like OA (HOA) and nitrogen-rich HOA (NHOA) factors linked with diesel, and compressed natural gas and petrol, respectively in Delhi. Interestingly, they also reported a significant increase in chloride concentration (522%) from pre-monsoon to post-monsoon seasons indicating large chlorine emissions from crop residue burning and open waste burning. Multiple studies carried out at various sites in Delhi reported interesting features of particulate matter properties. For example, Reyes-Villegas et al.^29^ used PMF to identify OA sources, Tobler et al.^35^ reported 39–49% OA contribution in total PM_2.5_ mass burden from December 2017 to May 2018 using ToF-ACSM. Source apportionment of NR-PM_2.5_ by Manchanda et al.^36^ during the COVID-19 lockdown in Delhi found changes in meteorological conditions to be responsible for secondary chloride and dust-related sources along with other POA instead of lockdown-induced reduced emissions and interestingly found ~ 95% increase in low-volatility oxygenated organic aerosols compared to pre-lockdown.

To gain further insights, we present analyses of nearly real-time measurements of NR-PM_1_ species in Delhi using an Aerosol Chemical Speciation Monitor (ACSM) focusing on source apportionment of OA performed with multilinear engine (ME-2) model via SoFi interface. We further argue that our analyses reduce the rotational ambiguity of traditional PMF models, potentially providing improved source profiles of the OA for our measurement period. Mathematical splitting of the solutions with a higher number of factors may result in erroneous PMF results^37^. A special focus on the oxidative nature of PM_1_ in the atmosphere, therefore, is given based on two distinct pollution episodes identified during our measurements.

## Methodology

### Site description

The sampling location is inside the Indian Meteorological Department (IMD), Lodhi Road, New Delhi (Lat 28.588° N, Lon 77.217° E). The site is coming under the national-capital region (NCR) Delhi. Delhi is the capital city of India, considered to be one of the most populated and polluted cities in the world. The sampling site was surrounded by many small-scale industries within a 25 km vicinity, including paper industries, pharmaceutical industries, recycling and waste management plants, and huge waste dumping sites responsible for the emission of various types of pollutants into the atmosphere^33,38^. The average temperature was 19 ± 5 °C for the entire sampling period, with a minimum and maximum value of 7.0 °C during nighttime and 31.8 °C during daytime, respectively. The relative humidity varied between 11–90% during the sampling period with an average value of 58 ± 21%. All the data of meteorological parameters were collected from the Centre Pollution Control Board (CPCB) operated station in Jawaharlal Nehru Stadium, which is less than 1.5 km from the campaign location. Delhi experiences a heavy pollution load during the winter months, with AQI reaching poor to very poor even after implementing many emission control strategies.

### Aerosol sampling and data analysis

The Aerodyne quadrupole Aerosol Chemical Speciation Monitor (q-ACSM; Aerodyne Research Inc., USA) is a modified version of the aerosol mass spectrometer (AMS) designed for long-term measurements of the major chemical composition of non-refractory submicron aerosol particles, including organic aerosol (OA), sulfate (SO_4_^2−^), nitrate (NO_3_^−^), ammonium (NH_4_^+^) and chloride (Cl^−^) in a high time resolution (about 15 min). The measurements were taken from 5th February to 3rd March 2018 in a temperature-controlled container with a sampling height of 5 m from the ground. The ambient air sample was drawn through a stainless tube at a flow rate of 3.085 L/min, out of which 85 cm^3^ was isokinetically allowed to pass into the ACSM sampling inlet. The sampled air was then allowed to pass through a silica gel dryer to minimize the impact of relative humidity (RH) on particle collection efficiency (CE). The sampling is done using jump scan mode with a scan rate of 200 amu/s in single scan mode.

The details of the operating principle and calibration of the instrument have been previously discussed^39,40^. Briefly, ultrafine particles with aerodynamic diameters 60–600 nm are sampled in a vacuum chamber through an aerodynamic lens. The PM is then flash-vaporized in a hot oven at around 600 °C and gets ionized at 70 eV, followed by subsequent detection using a quadrupole mass spectrometer. The transmission efficiency (TE) of the aerodynamic lens at the given condition was assumed to be at least greater than 50% for the selected size range. The concentrations of the chemical species are proportional to the measured ion signal.

Data processing has been done with the ACSM standard data analysis software (ACSM_local version 1.6.0.3 within the software package IGOR Pro (Wavemetrics, Inc., USA), using the same fragmentation table as AMS^41^. Elemental ratios O/C, H/C, and OM/OC have been calculated using the Improved-Ambient (I-A) method^42^.

### Instrument calibration

The absolute ionization efficiency (IEE), relative ionization efficiency (RIE), and response factor (RF were determined from the signal versus input mass plots of ammonium, nitrate, and sulfate using an aqueous solution of pure ammonium nitrate (NH_4_NO_3_) and ammonium sulfate ((NH4)_2_SO_4_). The aerosol particles have been generated with a TSI atomizer and dried by passing through a silica gel dryer, and then with a differential mobility analyzer (DMA 3081, TSI), the selected sized particles of 300 nm by an electrostatic classifier (3080, TSI) are allowed to enter into an aerosol diluter to control the concentration. The diluter output has been connected to the ½” ACSM inlet and then a condensation particle counter (CPC; 3776, TSI) to count the particle to the ½” Tee, where the ACSM bypass flow is connected. The same setup is used for NH_4_NO_3_ and then for (NH_4_)_2_SO_4_.

The RIE estimated for ammonium was 4.96 and sulfate was 0.9, with the default values for organic, nitrate, and chloride of 1.4, 1.1, and 1.3, respectively. In addition, sampling flow rate calibration, ionizer tuning, quadruple resolution adjustment, particle lens alignment, and mass-to-charge (m/z) calibration were performed at the beginning of the campaign.

Likewise, mass resolution, ionizer tuning and amplifier offset settings were conducted at the beginning and periodically to optimize the performance of the instrument. Besides, an effusive naphthalene source placed inside a stainless steel container with a pinhole leak of 1 µm in the vaporizer, which calibrates the instrument electronically with respect to temperature and pressure, also helps the calibration of ionization efficiency of the instrument^40^. A constant CE value of 0.5 was used for all the species, as discussed by Middlebrook et al.^43^, also used in Huffman et al.^44^ and Budisulistiorini et al.^45^. ACSM alternatively draws air samples through a filter (only gas) and without a filter (contains particles) and averages over ~ 15-min intervals for each measurement, minimizing gas interference during the analysis. The 3σ detection limits of the instrument for 30 min of averaging time for ammonium, organics, sulfate, nitrate, and chloride are 0.284 μg m^−3^, 0.148 μg m^−3^, 0.024 μg m^−3^, 0.010 μg m^−3^, and 0.011 μg m^−3^, respectively^40^. The higher detection limit of ammonium is due to its measurement on top of an extensive water and air background.

### Organic aerosol source characterization technique

This study focuses on source apportionment of OA in urban Delhi using the advanced receptor multilinear engine (ME-2) model with the source finder (SoFi) interface (v4.6, PSI)^37^ and unit mass resolution (UMR) data from ACSM. Major OA sources were identified using PMF and ME-2 applied through the SoFi interface^37,46,47^. Here, source apportionment with the ME-2 solver is carried out by constraining three primary OA factors, i.e., HOA (traffic), BBOA (biomass burning), and COA (cooking) using the a-value method and the two secondary factors were left free. This method uses prior information as user inputs to partially constrain one or more factor profiles. The values (a-value = 0.1, 0.2, 0.3….) decide the extent to which the results can vary from the original user input, hence providing a limit on the rotational ambiguity of the dataset over the unconstrained PMF. Detailed criteria for choosing the right number of factors are discussed in SI (Supplementary Figs. S1–S5). This approach was first introduced by Lanz et al.^46^ and implemented to OA measurements by Canonaco et al.^37^. ME-2 is specifically developed and has been used widely for source apportionment of OA obtained from AMS and ACSM measurements for the last decade^8,47–49^. The analysis resolved five different OA factors representing five different sources of OA emissions. Like many previous studies on ACSM measurements, ion signals beyond *m/z* 120 have been excluded from the source apportionment study due to the low signal-to-noise ratio and the unavoidable reasons described in Ng et al.^50^.

Depending upon organic and chloride concentrations observed during the campaign period and as categorized for the same campaign by Gunthe et al.^33^, we adopt the same classification here: (1) high organics and high chloride period referred to as pollution period 1 (PP1), and (2) high organic and low chloride period referred to as pollution period 2 (PP2). Briefly, days with daily average chloride mass loading ≥ 4 µg m^−3^ and organic mass concentration ≥ 40 µg m^−3^ were categorized under PP1. Conversely, PP2 was categorized based on the average chloride mass concentration < 2 µg m^−3^ with similar organic mass loading.

ME-2 analysis was performed separately for the entire campaign and different pollution periods (PP1 and PP2, detail discussed in "Episodic analysis of OA source apportionment"). Firstly, the PMF run has been made for a range of factors from two to eight with different seed runs (Supplementary Figs. S2–5). In this study, the five-factor solution was the most interpretable among all the unconstrained PMF solutions (see supplement section for details). Further separation of the factors led to the splitting of profiles with no enhancement in the data interpretation, whereas factor solution less than 5 showed a mixed profile. Several seed runs were made with different pseudorandom starts to obtain the most suitable solution after selecting the number of factors. The optimum factor solution was chosen by (1)-evaluating Q/Q_exp_ value, where Q is equal to the sum of squared residuals weighted by uncertainties from the measurements and model; Q_exp_ is the degree of freedom of the model solution; and (2)-the solution that minimizes the unexplained variance (Supplementary Fig. S1).

For this purpose, a solution was first obtained without a priori reference mass spectra (PMF run). Then previously originated mass spectra were introduced with varying a-value (degree of freedom) to constrain the solution spectra within a specific range. In this study, the factor profiles representing three primary sources such as hydrocarbon-like OA (HOA), biomass burning OA (BBOA), and cooking-like OA (COA) were constrained with varying a-values, based on the recommendation discussed in Canonaco et al.^37^. To obtain the precise factor solutions, several seed runs were carried out by constraining either HOA, BBOA, and COA at a time or separately with a-values ranging from 0.1 to 0.9 with steps of 0.1 for all the solutions. The optimal solutions were carefully chosen after the sensitivity test with (1) Q/Q_exp_ closer to one, (2) residual close to zero, and (3) observing the variations in the time series and daily patterns. The remaining two factors, so-called SOAs, were resolved by the unconstrained PMF run due to the explanation given in Canonaco et al.^51^ and Heringa et al.^52^. The use of factor profiles of other countries as constrain to apportion the emission sources of an Indian city like Delhi could introduce significant errors. Hence the target profile has been taken from a very recent study conducted at the same site in Delhi^10^. The resulting mass spectra of organics are compared with a group of compounds linked to known tracers by similar sources or processes to assign the respective sources precisely.

## Results and discussion

### Aerosol mass concentration and composition

**Fig. 1:**
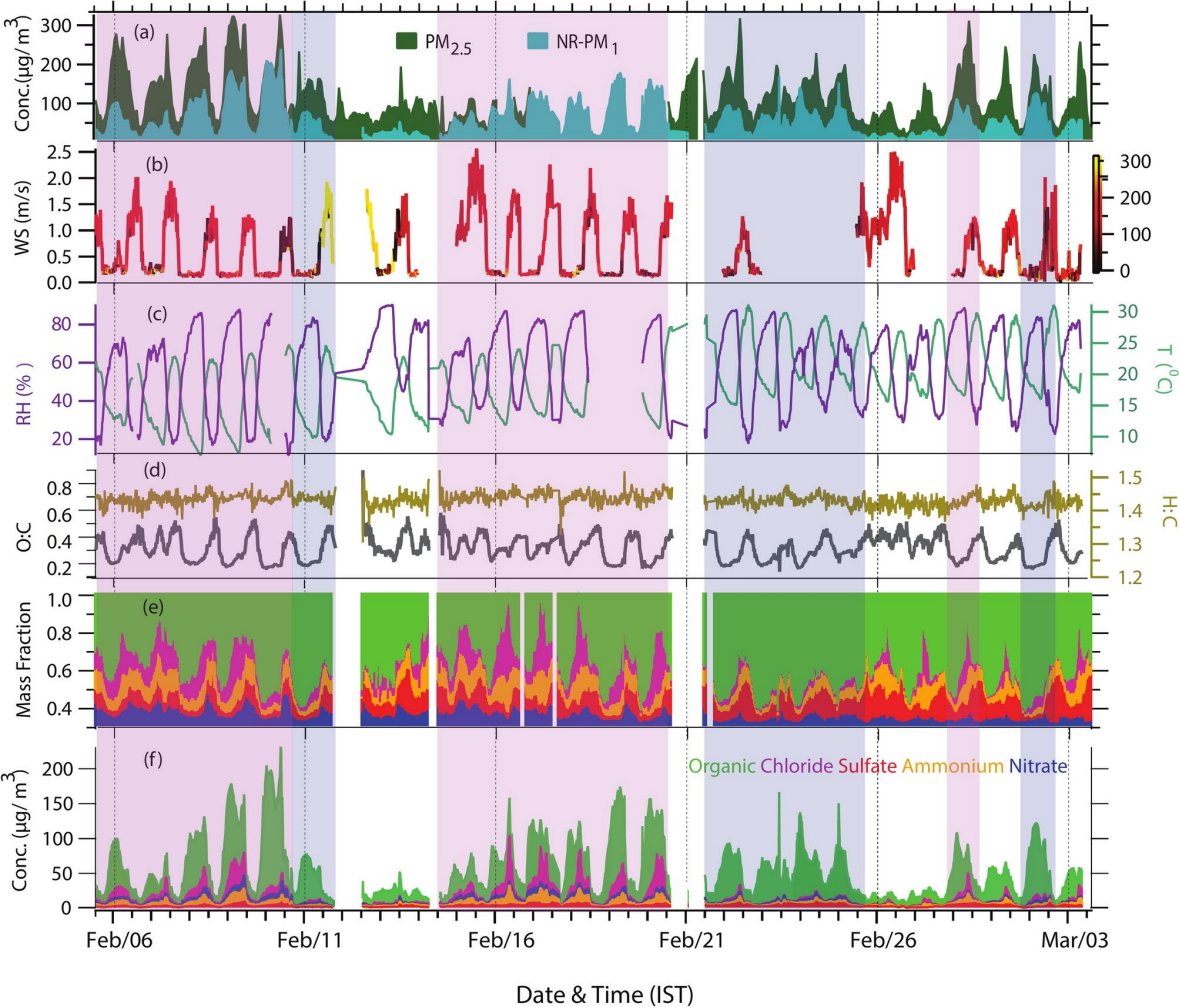
Time series of meteorological parameters and NR-PM_1_ components measured using Aerosol chemical speciation monitor (ACSM) during 5th February–3rd March 2018 in Delhi: (**a**) Mass concentration of PM_2.5_ obtained from US embassy (https://www.airnow.gov/international/us-embassies-and-consulates/) and total NR-PM_1_ measured by ACSM, (**b**) wind speed colour scaled by wind direction (**c**) relative humidity (RH) in purple and temperature (T) in green, (**d**) elemental ratio: O/C (black) and H/C (brown), (**e**) mass fraction of NR-PM_1_ components: organics, chloride, sulfate, ammonium, and nitrate in the same colour code as mentioned in (**f**) stacked plot of NR-PM_1_ components. The pink shaded area represents high organic high chloride days (PP1) and the high organic low chloride days are shown in blue shade.

**Fig. 2:**
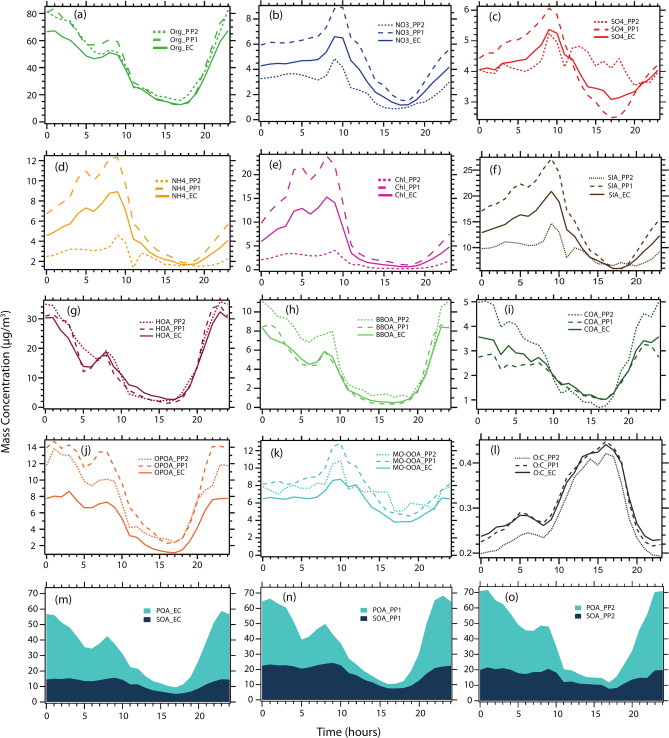
(**a**–**e**) show the average diel variation of NR-PM1 species and (**f**) diel of secondary inorganic aerosol (SIA = sulfate + nitrate + ammonium) during three different periods of interest: entire campaign (EC) period, high organic high chloride days (PP1), high organic low chloride days (PP2). Median daily pattern of five OA components obtained from ME-2 analysis are shown in (**g**–**k**), (**l**) O/C ratios. Median average daily pattern of POA and SOA during (**m**) EC (**n**) PP1, and (**o**) PP2.

The time-resolved mass concentration and chemical composition of NR-PM_1_ and the corresponding meteorological parameters from 5th February to 3rd March 2018 are shown in Fig. 1. The total NR-PM_1_ mass concentrations broadly varied from 7.7 to 238.5 µg m^−3^ during the study period, with an average (± standard deviation) of 58.0 ± 42.6 µg m^−3^. Overall, NR-PM_1_ mass was dominated by organics (69%) with average concentrations of 39.9 ± 32.2 µg m^−3^. Chloride contributed to the highest inorganic mass to the total NR-PM_1_ loading (5.9 ± 9.1 µg m^−3^) with a relative contribution of ~ 10%, as an exception to previous online measurements in urban areas of China, where sulfate is the dominant inorganic species^9,39,53^. Ammonium (4.6 ± 4.1 µg m^−3^) was the second-highest contributor to inorganic mass, followed by sulfate (3.9 ± 1.8 µg m^−3^) and nitrate (3.7 ± 2.9 µg m^−3^), making up 8%, 7%, and 6% fraction of the total NR-PM_1_ mass, respectively. The daily variation of NR-PM_1_ composition is shown in Fig. 2. Equilibrium between ammonia and hydrochloric acid (HCl) shifted towards the formation of ammonium chloride aerosol due to low temperature during winter, resulting in enhanced chloride concentration^54–56^. Such an underlying process, however, fails to explain the two- to three-fold increase in chloride mass during warmer seasons in Delhi^19^. The enhanced chloride concentration may result from increased HCl emissions near the sampling site, likely originating from metal processing industries, combustion of polyvinyl chloride, coal, biomass, plastic waste, and other anthropogenic and industrial activities influenced by local meteorological conditions^19,33^.

During the entire campaign period, including the PP1 and PP2 episodes, the measured ammonium correlated well (R^2^ = 0.88–0.92 and slope = 0.71–0.83) with ammonium predicted (ammonium required to neutralize the anionic species, namely sulfate, nitrate, and chloride) as shown in Supplementary Fig. S6, suggesting the robustness of our dataset. NH₄⁺ is predominantly formed through the gas-to-particle conversion of NH_3_, driven by its reactions with acidic precursors like H_2_SO_4_, HNO_3_, and HCl, resulting in the formation of (NH_4_)_2_SO_4_, NH_4_NO_3_, and NH_4_Cl^57,58^. The formation of NH_4_NO_3_ and NH_4_Cl is more pronounced under high humidity and low vapor pressure conditions, which enhance their atmospheric residence time compared to their gaseous precursors, including HNO_3_, HCl, and NH_3_^33,59^.

### Source apportionment of OA

**Fig. 3:**
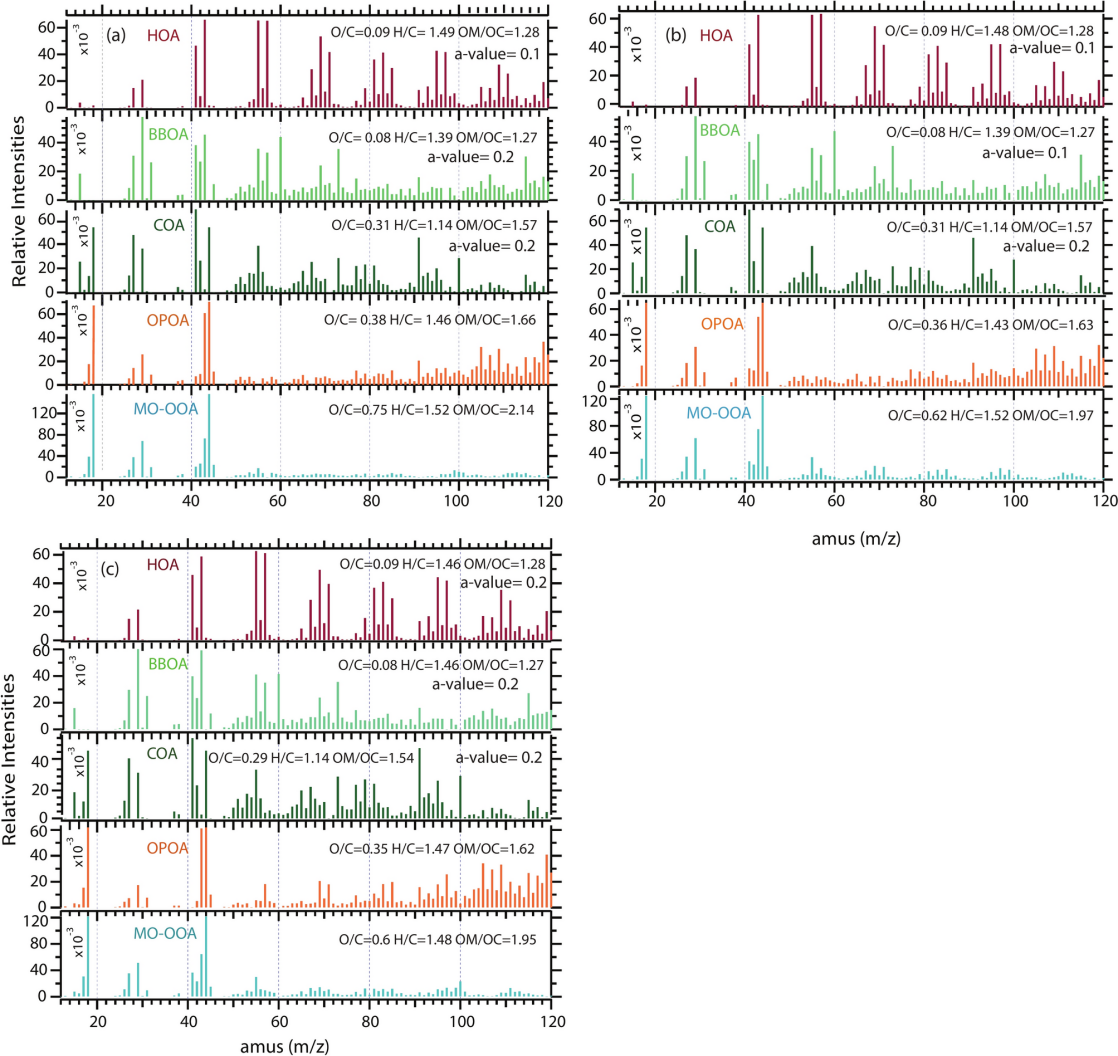
Mass spectra profile of five OA components resolved from ME-2 analysis separtely for all the period of interest: (**a**) EC, (**b**) PP1, (**c**) PP2.

**Fig. 4:**
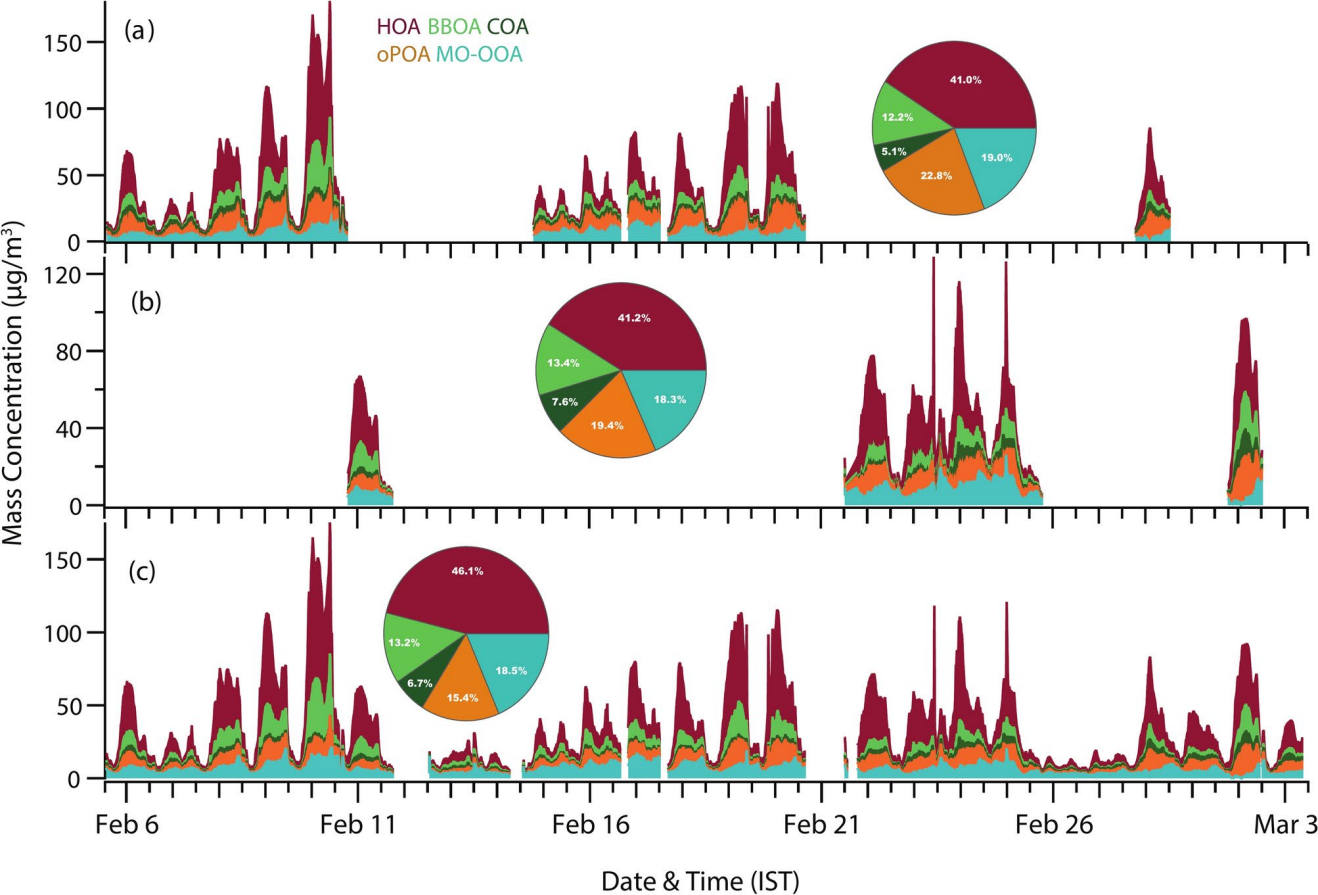
Time series of stacked OA factors (**a**) during PP1, (**b**) PP2, and (**c**) EC resolved from ME-2 Analysis. The pie charts in the respective panel show the relative contributions of OA components to the total OA mass.

Five major components of OA sources were deconvolved using ME-2 (Fig. 3) with three attributed to primary emissions, and two OA (OOA) as secondarily formed or oxidized primary aerosol particles indicating transformation and ageing due to long-range transport^60^. Traffic (HOA), biomass burning (BBOA), and cooking (COA) sources are identified as primary emissions, with an average total contribution of ~ 66% of the total organic mass during the entire study. In contrast, both the OOAs together make (34%), almost half of the total POA (Fig. 4). Mass spectra profiles, time series patterns, and diel variation of the individual factors were considered in assigning the factors. Below, we discuss the characteristic properties of each identified factor.

#### HOA

Primary emissions play a pivotal role in the ambient aerosol loading in urban environments. In Delhi, primary organic aerosols (POA) constitute ~ 66% of the total organic aerosol (OA) mass. In this study, HOA was the dominant primary organic factor, contributing an average of 46% to the total OA. Previous analysis studies of ambient aerosol have intensively identified HOA component, attributing it to primary sources such as the combustion of fossil fuel. These sources include n-alkanes, branched alkanes, cycloalkanes, and aromatics^4,61,62^. HOA is mainly dominated by hydrocarbons from the C_n_H_2n−1_ (*m/z* at 27, 41, 55, 69, 83, 97, and C_n_H_2n+1_ (*m/z* at 29, 43, 57, 71, 85, and 99)) families^47^. The HOA factor is often characterized by high-intensity signals at *m/z* 55 and 57. The campaign averages of O:C, H:C, and OM:OC ratios were 0.09, 1.49, and 1.28, respectively, consistent with typical POA values. HOA exhibited a distinct diel pattern, with concentrations increasing after sunset and peaking around midnight (~ 31 µg m^−3^), likely associated with the planetary boundary layer (PBL) height. A sharp peak between morning 7:00–9:00 corresponds to the local traffic rush during office hours.

#### BBOA

Open burning of wood and other biomass is a significant contributor to ambient OA globally^63–65^. In this study, BBOA accounted for ~ 13% of the total OA during the campaign period. BBOA showed strong correlations with known tracer ions for biomass-burning emitted aerosols, *m/z* 60 (R^2^ = 0.99) and 73 (R^2^ = 0.85), which are attributed to C_2_H_4_O_2_^+^ and C_3_H_5_O_2_^+^ respectively. These ions result from the pyrolysis of cellulose and similar high oxygen content polymers producing species such as levoglucosan (C_6_H_10_O_5_). Additional signals were observed at *m/z* 29, 31, 43, 55, 57, and 91 consistent with the other OA factors^66,67^. The O:C ratio of 0.08, H:C ratio of 1.39 and a strong signal at *m/z* 43 indicates the fresh emission. Biomass burning, particularly stubble burning in nearby agricultural fields (Punjab, Haryana, and Uttar Pradesh), is a major source of air quality degradation in the national capital region during winter and is a topic of ongoing scientific and political debate. Hence, BBOA in this study is likely derived from regional agricultural fires, with a minor contribution from biomass used for cooking in underdeveloped areas of the national capital. The diel profiles of BBOA reflected a similar pattern of HOA peaking at the highest concentration (~ 12 µg m^−3^) during nighttime.

#### COA

Studies have shown that COA is a common and crucial urban OA factor^49,68,69^. With 7% COA contribution was the lowest among all factors in total OA mass burden in Delhi. Characteristic peaks at *m/z* 55 (mostly C_3_H_3_O^+^ and/or C_4_H_7_^+^) and 57 (C_4_H_9_^+^ and/or C_3_H_5_O^+^) marked the presence of HOA and COA. The notable distinction between these two factors is identified based on the elevated *m/z* 55/57 intensity ratio observed in COA (7.1) in contrast to HOA (~ 1.0). In addition, a prominent peak at *m/z* 41 of COA indicates the presence of C_2_HO^+^ and C_3_H_5_^+^ ion groups (Fig. 3). These ratios were frequently considered as a strong marker for characterizing cooking-related OA components for AMS and ACSM measurements^68,70,71^. Trace of *m/z* 60 in the COA factor from the solid fuel and vegetables used during the cooking process was also predominantly observed during this study. The O:C and H:C ratios of COA were 0.3 and 1.14, respectively. The group of ions of *m/z* 41 and *m/z* 55 indicated the presence of unsaturated organic compounds like unsaturated fatty acids. However, our ME-2 result is also considered the hypothesis presented by Mohr et al.^72^ that the analysis of plastic or trash burning aerosols and cooking aerosol from ACSM data is likely to obtain a single component due to the resemblance of the unit-mass resolution (UMR) spectrums. The same hypothesis is also supported by the higher signal intensity at *m/z* 91 associated with C_7_H_7_^+^ ion group, mostly due to the fragmentation of aromatic compounds. In this study, the contribution from *f*_91_ and other higher *m/z* components is highest for COA, indicating PAH-related aerosol emission from the same kind of sources. Overall, the variation in COA factor could be due to several reasons, including differences in cooking methods, fuel type, and spices used, as well as the potential influence of other mixed sources. Interestingly, we have observed a very prominent signal at m/z 100 in COA for the first time, which could be used as a COA marker in future studies carefully.

#### OPOA and MO-OOA

Both SOA components together accounted for 34% of the total OA, with individual contribution of 15.4% and 18.6% for OPOA and MO-OOA, respectively. We partially interpret the term MO-OOA here to signify a higher contribution of compounds with shorter carbon chains and increased oxygen atom content. In contrast, OPOA likely corresponds to longer carbon chain compounds with lower oxygenation and higher volatility than MO-OOA. This interpretation is consistent with *m/z* peaks at 43 and 44 (attributed to C_2_H_3_O^+^and CO_2_^+^, respectively), primarily resulting from the decarboxylation of carboxylic acid^73^ showing higher O:C ratio than the fresh primary emissions. Notably, MO-OOA exhibits more oxidizing properties, evidenced by a higher *f*_44_/*f*_43_ ratio compared to OPOA, which is very consistent with previous studies^48,74,75^. Typically, SOA components correlate well with secondary inorganic species. The O:C and H:C ratios for OPOA were 0.38 and 1.46, lower than typical SV-OOA/ LO-OOA factor reported by Canagaratna et al.^42^, indicating relatively less oxidized SOA and lower atmospheric oxidative capacity (see "Oxidation process of OA"). This study recorded the highest O:C ratio of 0.75 for MO-OOA, indicating the highest oxidation level among the OA factors. However, in this study, we use the term MO-OOA, in place of "LV-OOA" and OPOA as "SV-OOA," originally introduced by Jimenez et al.^76^, as we were unable to precisely measure compound volatility in this campaign.

All three primary OA components, including HOA, BBOA, and COA, are directly emitted into the atmosphere as particles. Meanwhile, OPOA is considered to be oxidized POA formed either by evaporation and condensation mechanism or by heterogeneous oxidation in the atmosphere^77^. Here, OPOA could be associated with a specific burning source that could not be categorized under other sources, such as HOA, BBOA, or COA, but showed a notable correlation with Cl⁻. This source is likely linked to plastic burning, which may contribute to the oxidation of specific POA distinct from other primary sources^29,33^. MO-OOA forms due to gas-to-particle conversion characterized by reduced volatility and greater oxygenation through one or more chemical reactions. The earlier study reported that the elevated levels of non-refractory particulate chloride in Delhi are likely attributable to the gas-to-particle partitioning of HCl into aerosol water, facilitated by typical winter haze conditions characterized by high relative humidity (RH), low temperatures, and excess ammonia^33^. However, this process requires particles to persist in the atmosphere for extended periods, enabling potential long-range transport depending on meteorological conditions^77^. Overall, it may be noted that the presence of high chloride in the regional atmosphere of Delhi, represents the complex atmospheric chemistry scenario where, based on the previous studies, it is likely that high concentration of chloride can alter the oxidation pathways of SOA precursors and oxidation states of POA leading to higher SOA production and organohalogen productions^78^.

### Episodic analysis of OA source apportionment

The separate source apportionment for high organics and high chloride period (PP1) and high organic and low chloride period (PP2) of OA for better and accurate analysis of envisaging the likely sources of these OA was carried out (Supplementary Fig. S7). As mentioned, HOA dominated during both periods, with a relative contribution of > 40% to the total OA, and the contribution from BBOA to OA remained unchanged. However, COA contributed 3% more in OA mass burden during PP2 compared to PP1. The time series and relative contribution of the five components resolved for OA during the entire campaign and for PP1 and PP2 are depicted in Fig. 4. The overall SOA contributed 41.8% in PP1 as compared to 37.7% during PP2 (Supplementary Table S1). The relatively higher mass concentration of OPOA observed during PP1 (9.3 ± 4.7 µg m^−3^) than PP2 (7.8 ± 3.8 µg m^−3^) resulted in a 19% increase in its mass contribution between the two episodes. Higher atmospheric oxidative capacity owing to high chloride plausibly can explain such an increase in OPOA mass concentration during PP1. The increased signal intensity from higher *m/z* 77, 91 and 115 consistently in the OPOA profiles also implies the presence of PAH and long-chain aliphatic type high molecular weight compounds^79^. However, identification, characterization, and quantification of these components are beyond the scope of the instrument/study and need further investigations with instruments like high-resolution AMS.

Quantitatively, a good correlation between SOA and secondary inorganic species, mainly SO_4_^2−^ and NO_3_^−^, has been reported in many studies Zhang et al. and references therein^64^. In PP2, MO-OOA correlated well with SO_4_^2−^ (R^2^ = 0.69) and OPOA with NO_3_^−^ (R^2^ = 0.51) (Supplementary Fig. S8), consistent with the result reported by preceding studies, indicating both the components share a similar process of formation and evolution of these species^80^. Surprisingly, during PP1, MO-OOA showed a better correlation with NO_3_^−^ (R^2^ = 0.71) compared to SO_4_^2−^ (R^2^ = 0.56), and the correlation of NO_3_^−^ vs. OPOA dramatically lowered with an R^2^ value of 0.29 indicated a different formation mechanism of both the species. A similar kind of result has been reported in Zhang et al.^69^, where MO-OOA showed a similar time trend with NO_3_^−^ (R^2^ = 0.74), explaining the formation of the species due to photochemical production over gas-particle partitioning during lower temperature conditions. This observation infers that during the higher chloride episode, both NO_3_^−^ and OPOA probably follow different formation pathways (Supplementary Fig. S8). The enhanced OPOA concentration observed during PP1 may suggest the occurrence of atmospheric chloride reactions with OA, potentially leading to the formation of organohalogens or halogenated hydrocarbons, which are known to be highly toxic upon inhalation^80,81^. However, this hypothesis remains speculative in the present study due to the lack of experimental data and requires validation through dedicated research. Our previous research demonstrated that the aqueous-phase reaction of chloride with N_2_O_5_ during nighttime extends the NO_x_ lifetime by producing nitryl chloride (ClNO_2_). During daytime, ClNO_2_ undergoes photolysis, enhancing ozone and particulate matter formation through Cl atom pathways^33,81^. These findings highlight the importance of understanding chloride-induced processes in the atmosphere and their implications for air quality and human health. The experimental and computational studies that can be employed to discern the mechanistic roles of chloride in organic aerosol oxidation processes, including advanced mass spectrometry with higher mass resolution for effective identification of organohalogen formation in various atmospheric processes^82,83^.

The hygroscopicity parameter κ_chem_ estimated using simple mixing ratios of organics and inorganics as described in Gunthe et al.^84^ was found to be 0.26 (unitless) averaged over the entire study, which during PP1, increased to 0.40 and reduced to 0.28 during PP2. According to the literature, the hygroscopicity of OA is directly dependent on the oxidation level and increases with the aging of aerosol^85^, i.e., oxidized aerosols are more hygroscopic than the freshly emitted aerosol, which further supports well with the argument that the atmospheric oxidation process in Delhi is enhanced due to chlorine emissions.

#### Diel profile of OA factors

The daily pattern and relative contribution of the five different NR-PM_1_ species during the entire study and periods PP1 and PP2 are shown in Fig. 2a–e. A pronounced diel pattern was observed in all the identified components. On average, daytime concentrations peaked after 8:00 h during morning traffic rush hour and steadily decreased after 9:00 h, reaching their minimum at 17:00–19:00 h in the afternoon. The daily pattern of any ambient species can be explained as a combined result of many atmospheric phenomena, such as the accumulation of pollutants due to varying mixing height of the planetary boundary layer (PBL), secondary formations due to daytime photochemistry, local meteorology (wind speed, wind direction, temperature and RH) as well as primary emissions from local activities (traffic, construction, cooking, etc.).

All the OA factors showed pronounced diel variation throughout the campaign period. Figure 2g–k presents the daily average of the five resolved OA factors for both the periods and the entire study. HOA showed a very significant daily pattern with concentrations varying from 2.8 µg m^−3^ at 17:00 h, peaking at its highest value at 37.9 µg m^−3^ at 23:00 h. The average daytime OA value was around 50% lower than the nocturnal concentration level, which strongly demonstrates the importance of local anthropogenic emissions. Further, a peak around 8:00 h in the morning corresponded to the local rush hour, which drastically decreased after 9:00 h with a rise in the PBLH. We observed that the trend in diel variations of all three primary OA (HOA, BBOA and COA) showed an overall similar pattern. The enhanced diel pattern of these POAs with a peak in the early morning and late evening time, is mostly due to the resulting accumulation of pollutants, which is reduced during noontime due to the elevated boundary layer.

The daily variation of MO-OOA was relatively less pronounced compared to the other OA factors (Fig. 2k). The observed pattern was very much identical with secondary SO_4_^2−^ aerosol component (Fig. 2), indicating a direct association of sources between these two species. The daily pattern varied significantly during PP1 and PP2, and from other OA factors, indicating different formation pathways for the different periods. Previous studies conducted on source identification of ambient OA have shown an enhanced concentration of MO-OOA like aerosols during noontime corresponding to the photochemical process when sufficient sunlight is available^86–88^. In Delhi, the MO-OOA exhibited a peak around 9:00–10:00 h, consistent with the observations reported by Hua et al.^89^ in Beijing, China. However, unlike in Beijing, where MO-OOA concentrations remained elevated throughout the day, the concentrations in Delhi displayed a contrasting temporal pattern (Fig. 2k)^89^. In contrast, during PP2, MO-OOA showed the first peak between 9:00 and 10:00 h in the morning, with a considerable decrease that continued to peak till 17:00 h, i.e., throughout the daytime when the photochemistry is active. The distinct variation in MO-OOA, with a peak observed between 8:00 and 10:00 h, is noteworthy. We hypothesize that this peak results from the oxidation of precursors by chlorine, as other photochemical activities are likely minimal during this time. The second phase of oxidation started after photochemistry was active, indicating the widely reported photochemical oxidation process in the presence of OH radicals like previously reported LV-OOA factors. A similar kind of tri-modal peak of SOA has also been reported in Zhang et al.^88^, where the growth of the secondary particles was explained due to the condensation mechanism in the different size ranges. The hypothesis is that the oxidation processes in Delhi are significantly influenced by chloride in addition to OH radicals, unlike other urban locations across the world. This requires further validation with more specific and dedicated laboratory experiments replicating the atmospheric conditions of Delhi. However, the overall percentage contributions from the two combined SOA factors (OPOA + MO-OOA) (comprising about 14.5 and 15.1% in PP1 and PP2, respectively) were similar, indicating consistent availability of sufficient SOA precursors in the atmosphere.

The overall diel plots of all POA and SOA components exhibited distinct variability during both periods. However, no significant differences were observed in any OA components throughout the entire campaign as well as for PP1 and PP2 periods. Specifically, in PP1, the SOA concentration showed a pronounced daily pattern with an increased concentration during morning hours (Fig. 2m–o). However, the diel patterns exhibited weak variations during PP2 with slightly elevated concentration during the afternoon hours, reflecting enhanced photochemistry throughout this period. As discussed above, POA (= HOA + BBOA + COA) comprised ~ 66% of the total OA, whereas SOA made up only ~ 34%. Both the SOA together (= OPOA + MO-OOA) were well correlated with secondary inorganic aerosol (SIA) species (= sulfate + ammonia + nitrate) with an R^2^ value of 0.64 consistent with the result obtained from previous ACSM and AMS studies^90^.

#### Oxidation process of OA

**Fig. 5:**
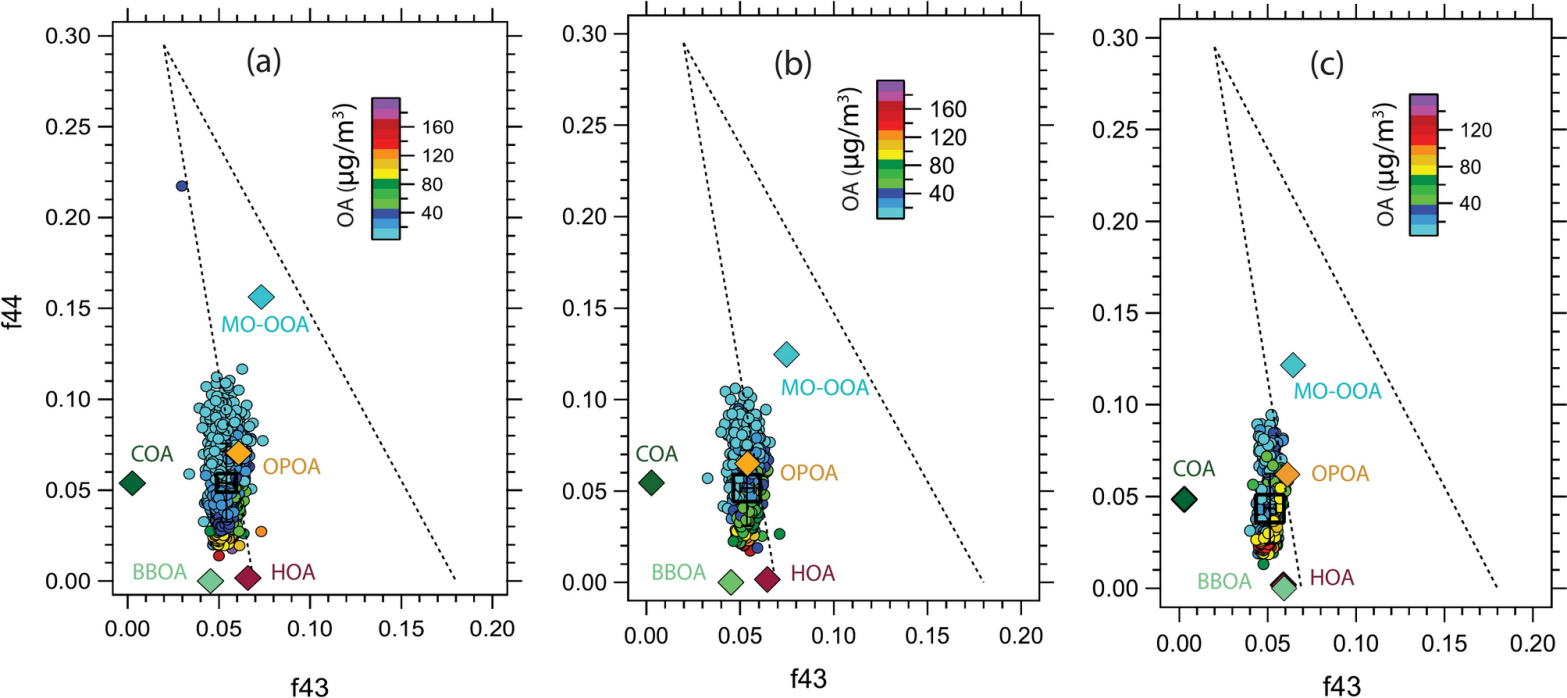
Plot of f44 vs f43 for (**a**) EC, (**b**) PP1, and (**c**) PP2 color coded by OA mass concentration. The corresponding OA components (HOA, BBOA, COA, OPOA, and MO-OOA) are placed as diamond markers and color-coded by OA type. The triangle area enclosed by two black dash lines and the bottom axis has been adopted from Ng et al. (2010).

The triangle plot of *f*_44_ vs. *f*_43_ has been extensively used in the ACSM community to characterize the extent of oxidation and atmospheric aging process of OA^48,91^. Figure 5 shows the behavior of *f*_43_ and *f*_44_ fragments acquired during the entire study period, PP1 and PP2, separately. Here, *f*_43_ and *f*_44_ represent the ratio of *m/z* 43 and 44 to the total OA signal, and the dotted line in the triangle-shape (known as Ng triangle), indicates the atmospheric oxidation process with the most aged OA placed in the top left corner and the fresh in the bottom right position^73^.

HOA and BBOA fall almost in the same region of the plot with similar aging levels and freshly produced aerosol particles. COA is positioned at a greater scale of *f*_44_ comparable with OPOA, though it has a lower *f*_43_ value, indicating a slightly higher oxidation level than the other two POA factors. The position of OPOA in the plot also categorizes it as a low-oxidized aerosol. MO-OOA falls in the highest fraction of *m/z* 44, representing a more oxidized, aged OA. Most importantly, all OAs points are less scattered/centered towards the left or outside of the triangle line during all the selected periods. The small *f*_44_ values, concentrated in the lower left corner, suggest that regardless of the precursor or oxidant present, there is a lack of substantial oxidation of OA in comparison to other urban areas documented. Hence, the particles are freshly emitted^91^, which is also consistent with the O/C ratio as well as the ME-2 analysis result. Our observations show an opposite trend, which is reported in urban areas of China^9,49^ where the points are much scattered and lie mostly within the triangle region, though it follows quite a similar pattern with the study from the eastern Mediterranean region^92^. A similar kind of result has also been observed by Heringa et al.^52^ from a chamber experiment where emissions from diesel and wood burning are shown towards the edge of the left side of the triangle. Further, *f*_43_ vs. *f*_44_ has been analyzed separately for daytime and nighttime during the campaign period (Supplementary Fig. S9). A small difference can be observed from the two figures, indicating a slightly higher oxidation rate during the daytime, with OA pointing towards a somewhat higher value of *f*_44_ than at nighttime.

There are many factors responsible for the oxidation of atmospheric aerosol. Conventionally, the most dominant oxidation pathway of most of the primary pollutants is driven by hydroxyl radicals. However, in Delhi, during the winter months, the production of hydroxyl radicals might be low due to the shorter duration of the sunlight and water vapor. Hence, the chemical transformation of primary pollutants to secondary is possibly slower due to insufficient availability of the oxidant in the atmosphere. Figure 1d shows the time series of O/C and H/C ratios for the study period. The average OM/OC ratio was 1.57 for the entire study. The same values for the O/C and H/C ratios were 0.31 and 1.43, respectively. The lower O/C and OM/OC ratios again indicate that OA in this study is overall less oxidized than the reported values from other urban areas^63^.

## Conclusions

We performed detailed source characterization of the ambient submicron particles in Delhi, identifying different pollution episodes. The average mean NR-PM_1_ concentration for the entire study was 58.0 ± 42.6 µg m^−3^, varied from 7.7 to 238.5 µg m^−3^, indicating the severity of PM pollution during the study period. Organics, chloride, ammonium, sulfate, and nitrate contributed 69%, 10%, 8%, 7%, and 6% fractions of the total NR-PM_1,_ respectively. The systematic source apportionment of the organics mass spectrum using the ME-2 model with a-value approach yielded five major factors identified as HOA (46%), BBOA (13%), COA (7%), OPOA (15%), and MO-OOA (19%). The POA (= HOA + BBOA + COA) contributed ~ 66% to the total organic mass loading, twofold higher than SOA (= OPOA + MO-OOA), indicating the dominance of freshly emitted anthropogenic emissions as major cause of the pollution episodes, which was further supported by pronounced diel variation of all the measured species.

Two different types of pollution episodes were categorized based on organic and chloride concentrations and analyzed separately for more insight into the sources. Out of the five resolved OA sources, HOA, which is considered a fingerprint for fossil fuel emissions, had a consistently dominant contribution during both the periods with a fraction of 40% of total OA mass, indicating higher pollution load mainly due to local vehicular emissions. PP1 was marked with a higher contribution of OPOA (23%), coinciding with higher chloride concentration compared to PP2 (19%), highlighting the possible influence of particulate chloride in secondary formation processes. Further, MO-OOA showed a significant difference between the daily variations during PP1 and PP2, implying possible differences in formation and evolution processes with weak daily variations during PP2 compared to pronounced daily variations during PP1.

All identified primary factors indicated pronounced diel variations with POA showing two fold nighttime increase in mass loading compared to daytime. The absolute concentration of MO-OOA was lower during the mid-day, unlike the results obtained from other urban locations clearly indicating the different formation pathways over this part of the world. The average O:C value of the entire campaign was 0.31, indicating OAs were overall poorly oxidized. The same has also been confirmed from the *f*44 vs. *f*43 triangle plot. Therefore, it is evident that the aerosols in Delhi are mixed of less-oxidized, freshly generated particles and secondarily formed highly-oxidized aged particles. However, further high-resolution, long-term measurements and dedicated field experiments as well as chamber experiments are immediately required to elucidate the organic composition in Delhi to better understand and delineate the contribution of primary and secondary in total PM mass burden for improved policy decisions for pollution abetment.

## Supplementary Information

Supplementary Information.

## Data Availability

All the source data are available in the figshare repository: 10.6084/m9.figshare.27301782 upon the publication of the manuscript.

## References

[1] Fuller, R. et al. Pollution and health: a progress update. *Lancet Planet. Health.* **6**, e535–e547 (2022). 35594895 10.1016/S2542-5196(22)00090-0 PMC11995256

[2] Ravindra, K. et al. Real-time monitoring of air pollutants in seven cities of North India during crop residue burning and their relationship with meteorology and transboundary movement of air. *Sci. Total Environ.* **690**, 717–729 (2019). 31301511 10.1016/j.scitotenv.2019.06.216

[3] Seinfeld, J. H., Pandis, S. N. & Noone, K. Atmospheric chemistry and physics: from air pollution to climate change. *Phys. Today*. **51** (1998).

[4] Zhang, Q. et al. Ubiquity and dominance of oxygenated species in organic aerosols in anthropogenically-influenced Northern Hemisphere midlatitudes. *Geophys. Res. Lett.* **34**, 1–6 (2007).

[5] Hallquist, M. *et al.* Hallquist et al., 2009. *Atmos. Chem. Phys.* **9**, 5155–5236 (2009).

[6] Lin, C. et al. Wintertime aerosol dominated by solid-fuel-burning emissions across Ireland: Insight into the spatial and chemical variation in submicron aerosol. *Atmos. Chem. Phys.* **19**, 14091–14106 (2019).

[7] Elser, M. et al. New insights into PM2.5 chemical composition and sources in two major cities in China during extreme haze events using aerosol mass spectrometry. *Atmos. Chem. Phys.* **16**, 3207–3225 (2016).

[8] Reyes-Villegas, E. et al. Organic aerosol source apportionment in London 2013 with ME-2: Exploring the solution space with annual and seasonal analysis. *Atmos. Chem. Phys.* **16**, 15545–15559 (2016).

[9] Guo, J. et al. Characterization of submicron particles by time-of-flight aerosol chemical speciation monitor (ToF-ACSM) during wintertime: Aerosol composition, sources, and chemical processes in Guangzhou. *China. Atmos. Chem. Phys.* **20**, 7595–7615 (2020).

[10] Reyes-Villegas, E. et al. PM1 composition and source apportionment at two sites in Delhi, India across multiple seasons. Atmos. Chem. Phys. Discuss. 10.5194/acp-2020-894 (2020).

[11] Zhao, J. et al. Organic aerosol processing during winter severe haze episodes in Beijing. *J. Geophys. Res. Atmos.* **124**, 10248–10263 (2019).

[12] Huang, X. F. et al. Exploration of PM2.5 sources on the regional scale in the Pearl River Delta based on ME-2 modeling. *Atmos. Chem. Phys.* **18**, 11563–11580 (2018).

[13] Pant, P. et al. Analysis of size-segregated winter season aerosol data from New Delhi, India. *Atmos. Pollut. Res.*10.1016/j.apr.2015.08.001 (2016).

[14] Trivedi, D. K., Ali, K. & Beig, G. Impact of meteorological parameters on the development of fine and coarse particles over Delhi. *Sci. Total Environ.* **478**, 175–183 (2014). 24531126 10.1016/j.scitotenv.2014.01.101

[15] Maji, S., Ghosh, S. & Ahmed, S. Association of air quality with respiratory and cardiovascular morbidity rate in Delhi, India. **3123**, (2018). 10.1080/09603123.2018.1487045 29963909

[16] Gadi, R., Shivani, S. S. K. & Mandal, T. K. Source apportionment and health risk assessment of organic constituents in fine ambient aerosols (PM2.5): A complete year study over National Capital Region of India. *Chemosphere* **221**, 583–596 (2019). 30665088 10.1016/j.chemosphere.2019.01.067

[17] Guttikunda, S. K. & Goel, R. Health impacts of particulate pollution in a megacity-Delhi, India. *Environ. Dev.* **6**, 8–20 (2013).

[18] Maji, S., Ahmed, S., Siddiqui, W. A. & Ghosh, S. Short term effects of criteria air pollutants on daily mortality in Delhi, India. *Atmos. Environ.* **150**, 210–219 (2017).

[19] Gani, S. et al. Submicron aerosol composition in the world’s most polluted megacity: The Delhi Aerosol Supersite study. *Atmos. Chem. Phys.* **19**, 6843–6859 (2019).

[20] Tiwari, S. et al. Variability in atmospheric particulates and meteorological effects on their mass concentrations over Delhi, India. *Atmos. Res.* **145–146**, 45–56 (2014).

[21] Pant, P. & Harrison, R. M. Estimation of the contribution of road traffic emissions to particulate matter concentrations from field measurements: A review. *Atmos. Environ.* **77**, 78–97 (2013).

[22] Mishra, S. K. et al. Morphology of atmospheric particles over semi-arid region (Jaipur, Rajasthan) of India: Implications for optical properties. *Aerosol Air Qual. Res.* **15**, 974–984 (2015).

[23] Mandal, P. et al. High seasonal variation of atmospheric C and particle concentrations in Delhi. *India. Environ. Chem. Lett.* **12**, 225–230 (2014).

[24] Gorai, A. K., Tchounwou, P. B., Biswal, S. S. & Tuluri, F. Spatio-Temporal Variation of Particulate Matter(PM2.5) Concentrations and Its Health Impacts in a Mega City, Delhi in India. *Environ. Health Insights.* **12**, (2018). 10.1177/1178630218792861 PMC6102754 30147329

[25] Panda, S. *et al.* Organic and elemental carbon variation in PM2.5 over megacity Delhi and Bhubaneswar, a semi-urban coastal site in India. *Nat. Hazards.* **80**, 1709–1728 (2016).

[26] Saxena, M. *et al.* Water soluble inorganic species of PM10 and PM2.5 at an urban site of Delhi, India: Seasonal variability and sources. In *Atmospheric Research*, vol. 184 (Elsevier B.V., 2017).

[27] Sharma, S. K. et al. Source apportionment of PM10 by using positive matrix factorization at an urban site of Delhi, India. *Urban Clim.* **10**, 656–670 (2014).

[28] Singhai, A., Habib, G., Raman, R. S. & Gupta, T. Chemical characterization of PM1.0 aerosol in Delhi and source apportionment using positive matrix factorization. *Environ. Sci. Pollut. Res.* **24**, 445–462 (2017). 10.1007/s11356-016-7708-8 27726085

[29] Reyes-Villegas, E. et al. PM1 composition and source apportionment at two sites in Delhi, India, across multiple seasons. *Atmos. Chem. Phys.* **21**, 11655–11667 (2021).

[30] Kumar, V. et al. Highly time-resolved chemical speciation and source apportionment of organic aerosol components in Delhi, India, using extractive electrospray ionization mass spectrometry. *Atmos. Chem. Phys.* **22**, 7739–7761 (2022).

[31] Singh, A. *et al.* Sources and characteristics of light-absorbing fine particulates over Delhi through the synergy of real-time optical and chemical measurements. *Atmos. Environ.* **252**, (2021).

[32] Bhandari, S. et al. Sources and atmospheric dynamics of organic aerosol in New Delhi, India: Insights from receptor modeling. *Atmos. Chem. Phys.* **20**, 735–752 (2020).

[33] Gunthe, S. S. et al. Enhanced aerosol particle growth sustained by high continental chlorine emission in India. *Nat. Geosci.* **14**, 77–84 (2021).

[34] Patel, K. et al. Sources and dynamics of submicron aerosol during the autumn onset of the air pollution season in Delhi. *India.*10.1021/acsearthspacechem.0c00340 (2021).

[35] Tobler, A., Bhattu, D., Canonaco, F., Lalchandani, V. & Shukla, A. Chemical characterization of PM2.5 and source apportionment of organic aerosol in New Delhi, India Science of the Total Environment Chemical characterization of PM 2.5 and source apportionment of organic aerosol in New Delhi, India. 10.1016/j.scitotenv.2020.140924 (2020). 10.1016/j.scitotenv.2020.140924 32738681

[36] Manchanda, C., Kumar, M., Singh, V., Faisal, M. & Hazarika, N. Variation in chemical composition and sources of PM 2.5 during the COVID-19 lockdown in Delhi. *Environ. Int.* **153**, 106541 (2021). 33845290 10.1016/j.envint.2021.106541

[37] Canonaco, F., Crippa, M., Slowik, J. G., Baltensperger, U. & Prévôt, A. S. H. SoFi, an IGOR-based interface for the efficient use of the generalized multilinear engine (ME-2) for the source apportionment: ME-2 application to aerosol mass spectrometer data. *Atmos. Meas. Tech.* **6**, 3649–3661 (2013).

[38] S. Raj, S. *et al.* Planetary boundary layer height modulates aerosol—water vapor interactions during winter in the megacity of Delhi. *J. Geophys. Res. Atmos.* **126**, (2021).

[39] Sun, Y. et al. Characterization of summer organic and inorganic aerosols in Beijing, China with an Aerosol Chemical Speciation Monitor. *Atmos. Environ.* **51**, 250–259 (2012).

[40] Ng, N. L. et al. An Aerosol Chemical Speciation Monitor (ACSM) for routine monitoring of the composition and mass concentrations of ambient aerosol. *Aerosol. Sci. Technol.* **45**, 780–794 (2011).

[41] Allan, J. D. et al. A generalised method for the extraction of chemically resolved mass spectra from Aerodyne aerosol mass spectrometer data. *J. Aerosol. Sci.* **35**, 909–922 (2004).

[42] Canagaratna, M. R. et al. Elemental ratio measurements of organic compounds using aerosol mass spectrometry: Characterization, improved calibration, and implications. *Atmos. Chem. Phys.* **15**, 253–272 (2015).

[43] Middlebrook, A. M., Bahreini, R., Jimenez, J. L. & Canagaratna, M. R. Evaluation of composition-dependent collection efficiencies for the Aerodyne aerosol mass spectrometer using field data. *Aerosol. Sci. Technol.* **46**, 258–271 (2012).

[44] Huffman, J. A. et al. Chemically-resolved aerosol volatility measurements from two megacity field studies. *Atmos. Chem. Phys.* **9**, 7161–7182 (2009).

[45] Budisulistiorini, S. H. et al. Seasonal characterization of submicron aerosol chemical composition and organic aerosol sources in the southeastern United States: Atlanta, Georgia, and Look Rock, Tennessee. *Atmos. Chem. Phys.* **16**, 5171–5189 (2016).

[46] Lanz, V. A. et al. Source attribution of submicron organic aerosols during wintertime inversions by advanced factor analysis of aerosol mass spectra. *Environ. Sci. Technol.* **42**, 214–220 (2008). 18350899 10.1021/es0707207

[47] Wang, Y. C. *et al.* Chemical composition, sources and secondary processes of aerosols in Baoji city of northwest China. **158**, 128–137 (2017).

[48] Zhu, Q. et al. Improved source apportionment of organic aerosols in complex urban air pollution using the multilinear engine (ME-2). *Atmos. Meas. Tech.* **11**, 1049–1060 (2018).

[49] Sun, Y. *et al.* Source apportionment of organic aerosol from two-year highly time-resolved measurements by an aerosol chemical speciation monitor in Beijing, China. *Atmos. Chem. Phys. Discuss.* 1–33 (2018) 10.5194/acp-2017-1195.

[50] Ng, N. L. et al. Real-time methods for estimating organic component mass concentrations from aerosol mass spectrometer data. *Environ. Sci. Technol.* **45**, 910–916 (2011). 21186814 10.1021/es102951k

[51] Canonaco, F., Slowik, J. G., Baltensperger, U. & Prévôt, A. S. H. Seasonal differences in oxygenated organic aerosol composition: Implications for emissions sources and factor analysis. *Atmos. Chem. Phys.* **15**, 6993–7002 (2015).

[52] Heringa, M. F. et al. A new method to discriminate secondary organic aerosols from different sources using high-resolution aerosol mass spectra. *Atmos. Chem. Phys.* **12**, 2189–2203 (2012).

[53] Sun, Y. et al. Primary and secondary aerosols in Beijing in winter: Sources, variations and processes. *Atmos. Chem. Phys.* **16**, 8309–8329 (2016).

[54] Acharja, P. et al. Enhanced secondary aerosol formation driven by excess ammonia during fog episodes in Delhi, India. *Chemosphere* **289**, 133155 (2022). 34875290 10.1016/j.chemosphere.2021.133155

[55] Pawar, P. V. et al. Chloride (HCl / Cl-) dominates inorganic aerosol formation from ammonia in the Indo-Gangetic Plain during winter: modeling and comparison with observations. *Atmos. Chem. Phys.* **23**, 41–59 (2023).

[56] Pio, C. A. & Harrison, R. M. The equilibrium of ammonium chloride aerosol with gaseous hydrochloric acid and ammonia under tropospheric conditions. *Atmos. Environ.* **21**, 1243–1246 (1987).

[57] Singh, S. & Kulshrestha, U. C. Abundance and distribution of gaseous ammonia and particulate ammonium at Delhi, India. *Biogeosciences* **9**, 5023–5029 (2012).

[58] Gopinath, A. K. *et al.* Complex interplay between organic and secondary inorganic aerosols with ambient relative humidity implicates the aerosol liquid water content over India during wintertime. *J. Geophys. Res. Atmos.* **127**, (2022).

[59] Acharja, P. et al. Thermodynamical framework for effective mitigation of high aerosol loading in the Indo-Gangetic Plain during winter. *Sci. Rep.* **13**, 1–10 (2023). 37608151 10.1038/s41598-023-40657-w PMC10444748

[60] Mohr, C. et al. Characterization of primary organic aerosol emissions from meat cooking, trash burning, and motor vehicles with high-resolution aerosol mass spectrometry and comparison with ambient and chamber observations. *Environ. Sci. Technol.* **43**, 2443–2449 (2009). 19452899 10.1021/es8011518

[61] Canagaratna, M. R. et al. Chase studies of particulate emissions from in-use New York City vehicles. *Aerosol Sci. Technol.* **38**, 555–573 (2004).

[62] Ulbrich, I. M., Canagaratna, M. R., Zhang, Q., Worsnop, D. R. & Jimenez, J. L. Interpretation of organic components from positive matrix factorization of aerosol mass spectrometric data. *Atmos. Chem. Phys.* **9**, 2891–2918 (2009).

[63] Bougiatioti, A. et al. Processing of biomass-burning aerosol in the eastern Mediterranean during summertime. *Atmos. Chem. Phys.* **14**, 4793–4807 (2014).

[64] Zhang, Q. et al. Understanding atmospheric organic aerosols via factor analysis of aerosol mass spectrometry: A review. *Anal. Bioanal. Chem.* **401**, 3045–3067 (2011). 21972005 10.1007/s00216-011-5355-y PMC3217143

[65] Rattanavaraha, W. et al. Source apportionment of submicron organic aerosol collected from Atlanta, Georgia, during 2014–2015 using the aerosol chemical speciation monitor (ACSM). *Atmos. Environ.* **167**, 389–402 (2017).

[66] Alfarra, M. R. et al. Identification of the mass spectral signature of organic aerosols from wood burning emissions. *Environ. Sci. Technol.* **41**, 5770–5777 (2007). 17874785 10.1021/es062289b

[67] Lanz, V. A. et al. Source apportionment of submicron organic aerosols at an urban site by factor analytical modelling of aerosol mass spectra. *Atmos. Chem. Phys.* **7**, 1503–1522 (2007).

[68] Sun, Y. L. et al. Aerosol composition, sources and processes during wintertime in Beijing, China. *Atmos. Chem. Phys.* **13**, 4577–4592 (2013).

[69] Zhang, Y. et al. Chemical composition, sources and evolution processes of aerosol at an urban site in Yangtze River Delta, China during wintertime. *Atmos. Environ.* **123**, 339–349 (2015).

[70] Lanz, V. A. et al. Characterization of aerosol chemical composition with aerosol mass spectrometry in Central Europe: An overview. *Atmos. Chem. Phys.* **10**, 10453–10471 (2010).

[71] Crippa, M. et al. Identification of marine and continental aerosol sources in Paris using high resolution aerosol mass spectrometry. *J. Geophys. Res. Atmos.* **118**, 1950–1963 (2013).

[72] Mohr, C. et al. Identification and quantification of organic aerosol from cooking and other sources in Barcelona using aerosol mass spectrometer data. *Atmos. Chem. Phys.* **12**, 1649–1665 (2012).

[73] Ng, N. L. et al. Organic aerosol components observed in Northern Hemispheric datasets from Aerosol Mass Spectrometry. *Atmos. Chem. Phys.* **10**, 4625–4641 (2010).

[74] Minguillón, M. C. et al. Chemical characterization of submicron regional background aerosols in the western Mediterranean using an Aerosol Chemical Speciation Monitor. *Atmos. Chem. Phys.* **15**, 6379–6391 (2015).

[75] Reyes-Villegas, E. et al. Simultaneous aerosol mass spectrometry and chemical ionisation mass spectrometry measurements during a biomass burning event in the UK: Insights into nitrate chemistry. *Atmos. Chem. Phys.* **18**, 4093–4111 (2018).

[76] Jimenez, J. L. *et al.* Evolution of organic aerosols in the atmosphere. *Science (80-.).* **326**, 1525–1529 (2009). 10.1126/science.1180353 20007897

[77] Hildebrandt, L. et al. Aged organic aerosol in the Eastern Mediterranean: The Finokalia Aerosol Measurement Experiment-2008. *Atmos. Chem. Phys.* **10**, 4167–4186 (2010).

[78] Li, Q. et al. Halogens enhance haze pollution in China. *Environ. Sci. Technol.* **55**, 13625–13637 (2021). 34591460 10.1021/acs.est.1c01949 PMC8529710

[79] Cash, J. M. *et al.* Seasonal analysis of submicron aerosol in Old Delhi using high resolution aerosol mass spectrometry: Chemical characterisation, source apportionment and new marker identification. 1–42 (2020).

[80] Decarlo, P. F. et al. Investigation of the sources and processing of organic aerosol over the Central Mexican Plateau from aircraft measurements during MILAGRO. *Atmos. Chem. Phys.* **10**, 5257–5280 (2010).

[81] Thornton, J. A. et al. A large atomic chlorine source inferred from mid-continental reactive nitrogen chemistry. *Nature* **464**, 271–274 (2010). 20220847 10.1038/nature08905

[82] Glasius, M. & Goldstein, A. H. Recent discoveries and future challenges in atmospheric organic chemistry. *Environ. Sci. Technol.* **50**, 2754–2764 (2016). 26862779 10.1021/acs.est.5b05105

[83] Laskin, A. et al. Mass spectrometric approaches for chemical characterisation of atmospheric aerosols: critical review of the most recent advances. *Environ. Chem*. **9**, 163–189.

[84] Gunthe, S. S. et al. Cloud condensation nuclei (CCN) from fresh and aged air pollution in the megacity region of Beijing. *Atmos. Chem. Phys.* **11**, 11023–11039 (2011).

[85] Kuang, Y. *et al.* Distinct diurnal variation of organic aerosol hygroscopicity and its relationship with oxygenated organic aerosol. *Atmos. Chem. Phys. Discuss.* 1–33 (2019) 10.5194/acp-2019-633.

[86] Sun, C. et al. Continuous measurements at the urban roadside in an Asian megacity by Aerosol Chemical Speciation Monitor (ACSM): Particulate matter characteristics during fall and winter seasons in Hong Kong. *Atmos. Chem. Phys.* **16**, 1713–1728 (2016).

[87] Xu, J. et al. Wintertime organic and inorganic aerosols in Lanzhou, China: Sources, processes, and comparison with the results during summer. *Atmos. Chem. Phys.* **16**, 14937–14957 (2016).

[88] Zhang, Q., Worsnop, D. R., Canagaratna, M. R. & Jimenez, J. L. Hydrocarbon-like and oxygenated organic aerosols in Pittsburgh: Insights into sources and processes of organic aerosols. *Atmos. Chem. Phys.* **5**, 3289–3311 (2005).

[89] Hua, Y. et al. Characteristics and sources of aerosol pollution at a polluted rural site southwest in Beijing, China. *Sci. Total Environ.* **626**, 519–527 (2018). 29353791 10.1016/j.scitotenv.2018.01.047

[90] Huang, X. F. et al. Highly time-resolved chemical characterization of atmospheric submicron particles during 2008 Beijing Olympic games using an aerodyne high-resolution aerosol mass spectrometer. *Atmos. Chem. Phys.* **10**, 8933–8945 (2010).

[91] Ng, N. L. et al. Changes in organic aerosol composition with aging inferred from aerosol mass spectra. *Atmos. Chem. Phys.* **11**, 6465–6474 (2011).

[92] Hildebrandt, L. et al. Sources and atmospheric processing of organic aerosol in the Mediterranean: Insights from aerosol mass spectrometer factor analysis. *Atmos. Chem. Phys.* **11**, 12499–12515 (2011).

