# Supplementary material for: Exploring the chemical composition and processes of submicron aerosols in Delhi using aerosol chemical speciation monitor driven factor analysis
Source: Sci Rep. 2025 Apr 24;15:14383. doi: 10.1038/s41598-025-99245-9 (PMC12022066; doi:10.1038/s41598-025-99245-9)
Supplement: Supplementary file 1 — Supplementary Information. [file 41598_2025_99245_MOESM1_ESM.docx]

**Supplement of: Exploring the chemical composition and processes of submicron aerosols in Delhi using the Aerosol Chemical Speciation Monitor driven factor analysis.**

Upasana Panda^1,2,a^, Supriya Dey^1,3,*^, Amit Sharma^4^, Aishwarya Singh^1,3^, Ernesto Reyes-Villegas^5,6^, Eoghan Darbyshire^5,7^, Samara Carbone^8^, Trupti Das^2^, James Allan^5,9^, Gordon McFiggans^5^, R. Ravikrishna^10,3^, Hugh Coe^5^, Pengfei Liu^11^ and Sachin S. Gunthe^1,3,*^

^1^EE Division, Department of Civil Engineering, Indian Institute of Technology Madras, Chennai, India.

^2^Department of Environment and Sustainability, CSIR –Institute of Minerals and Materials Technology, Bhubaneswar, India.

^3^Centre for Atmospheric and Climate Sciences, Indian Institute of Technology Madras, Chennai, India.

^4^Department of Civil and Infrastructure Engineering, Indian Institute of Technology Jodhpur, Karwar, Jodhpur, India.

^5^Department of Earth and Environmental Sciences, School of Natural Sciences, University of Manchester, Manchester, UK.

^6^Tecnologico de Monterrey, Escuela de Ingeniería y Ciencias, Av. General Ramon Corona 2514, Nuevo México, Zapopan CP 45138 Jalisco, Mexico.

^7^The Conflict and Environment Observatory, Hebden Bridge, West Yorkshire, UK.

^8^Institute of Agrarian Sciences, Federal University of Uberlândia, Uberlândia-MG, Brazil.

^9^National Centre for Atmospheric Science, University of Manchester, Manchester, UK.

^10^Department of Chemical Engineering, Indian Institute of Technology Madras, Chennai, India.

^11^School of Earth and Atmospheric Sciences, Georgia Institute of Technology, Atlanta, GA, USA.

^a^Present address: Kalinga Institute of Industrial Technology – Deemed to be University, Bhubaneswar, India.

**Correspondence to: Supriya Dey** ([supriya.dey@cacs.iitm.ac.in](mailto:supriya.dey@cacs.iitm.ac.in)) and **Sachin S. Gunthe** (s.gunthe@iitm.ac.in)

**1. Criteria for choosing factor solution**

The input file for PMF analysis (organics and organics error matrix) has been extracted from the default ACSM data analysis software by a six-step automated procedure. At first, the PMF run was made for a range of factors from two to eight. Then, to examine the stability of each solution, the algorithm was reset with varying initial conditions (seed runs). The most stable solution was observed for a five-factor solution with not much variation in Q/Qexp values. Once the factor solution was finalized, thirty seed runs were made to select the optimal solution. Then, the contribution of each factor to the total organic mass, with minimal unexplained mass, was observed, and finally, the variation of each factor compared and unexplained variation was less than 10% (Figure S1).


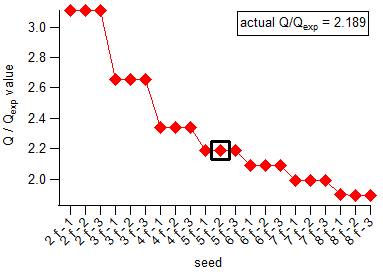

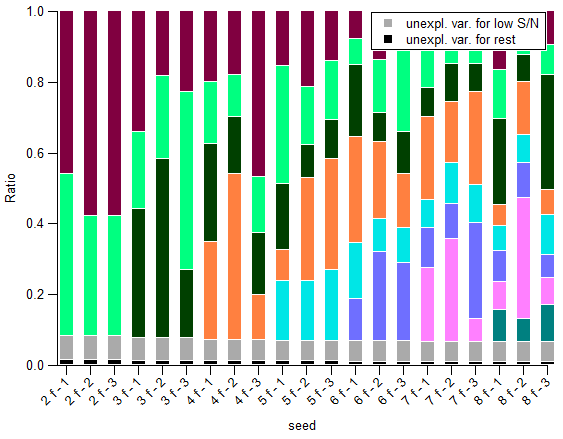


*Figure S1. Q/Qexp for different numbers of factors and seed run (left) variable plots for selecting the number of factor solutions (right)*

Figure S (2-5) represent the mass spectra and time series for two-factor, three-factor, four-factor, and six-factor PMF solutions. The factor solutions were selected mainly based on the mass spectra profile and correlation between the time series and various external tracers. Both two and three-factor solutions give mixed profiles for POA and OOA with higher contributions from m/z 44 and m/z 43 (Figure S2, S3). The four-factor solution gives a mixed profile for COA and OPOA, allowing further separation of the spectra (Figure S4). The six-factor solution starts splitting the mass spectra (Figure S5), COA and OPOA factors into three factors without providing a meaningful solution. So, we chose five as an optimum number of factors and analyzed them further to get the final source profile. Variation in fpeak had little impact on Q/Qexp and hence kept its default value as zero. More details about the raw data and analysis is given in Panda et al., (2024)


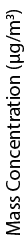

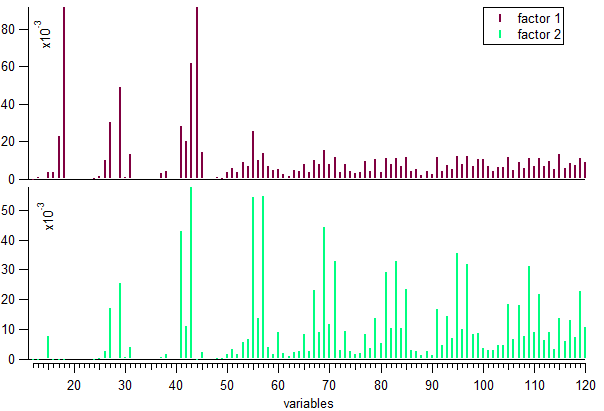

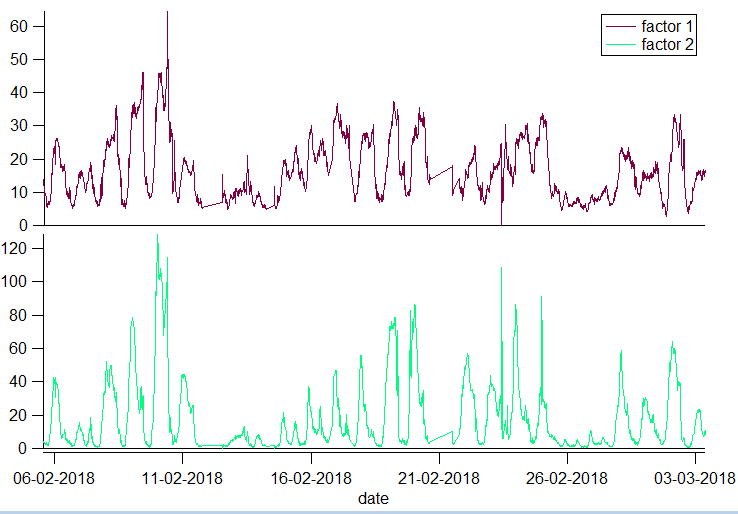

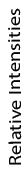


*Figure S2. Mass spectra and time series of two-factor PMF solution for seed run.*


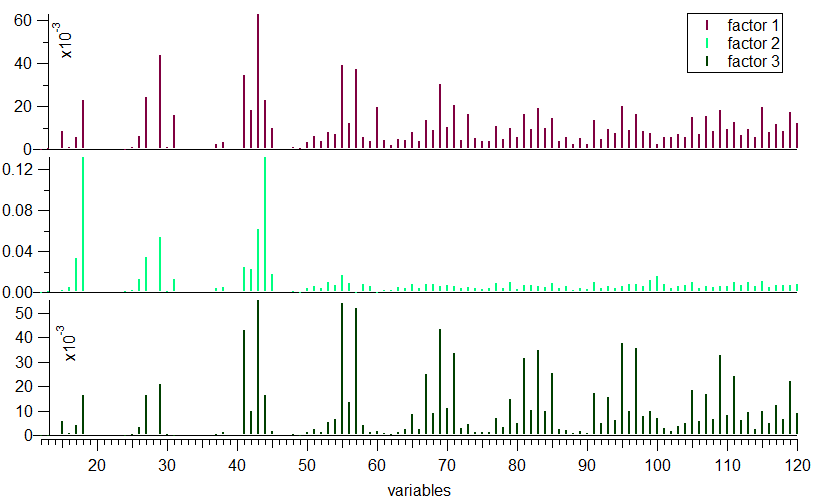

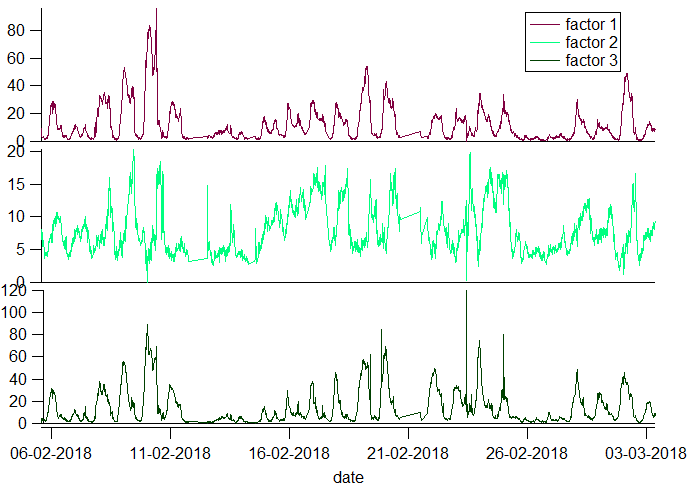

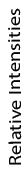

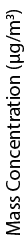


*Figure S3. Mass spectra and time series of three-factor PMF solution for seed run.*


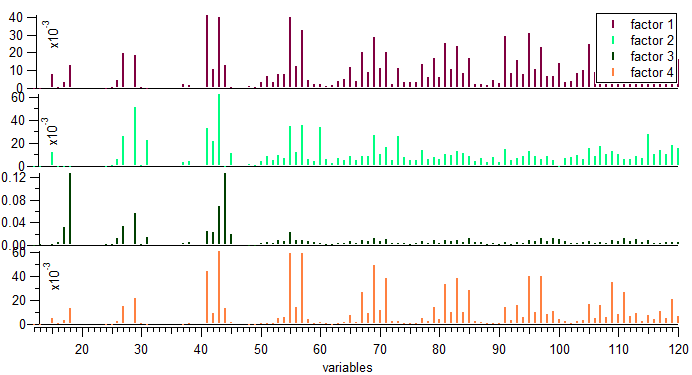

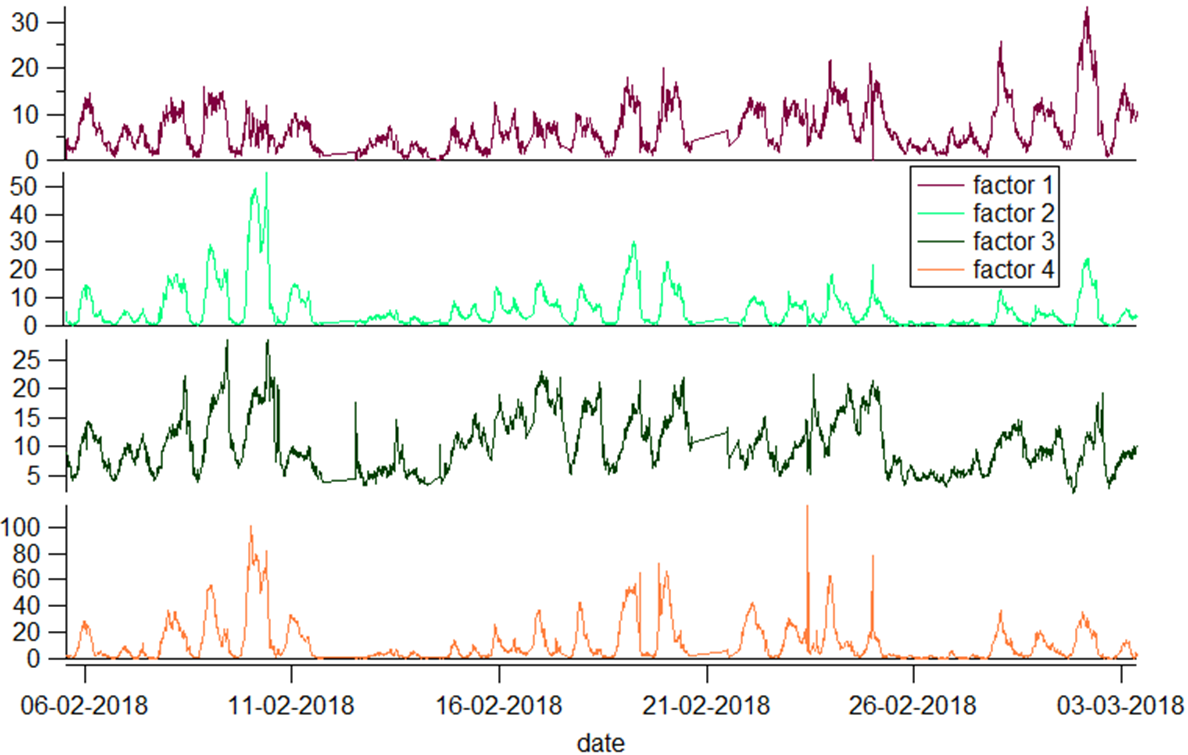

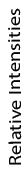

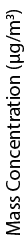


*Figure S4. Mass spectra and time series of four-factor PMF solution for seed run.*


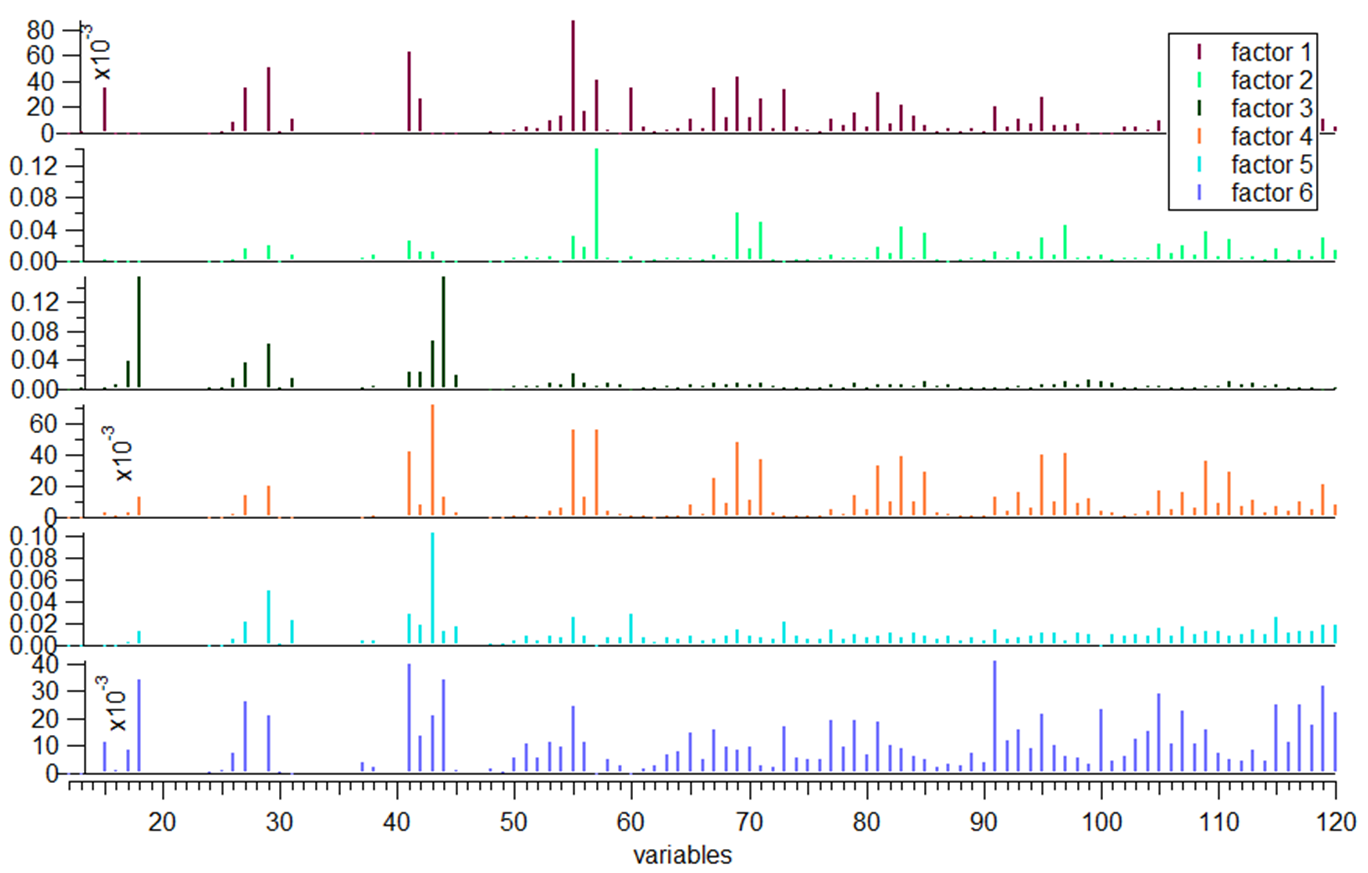

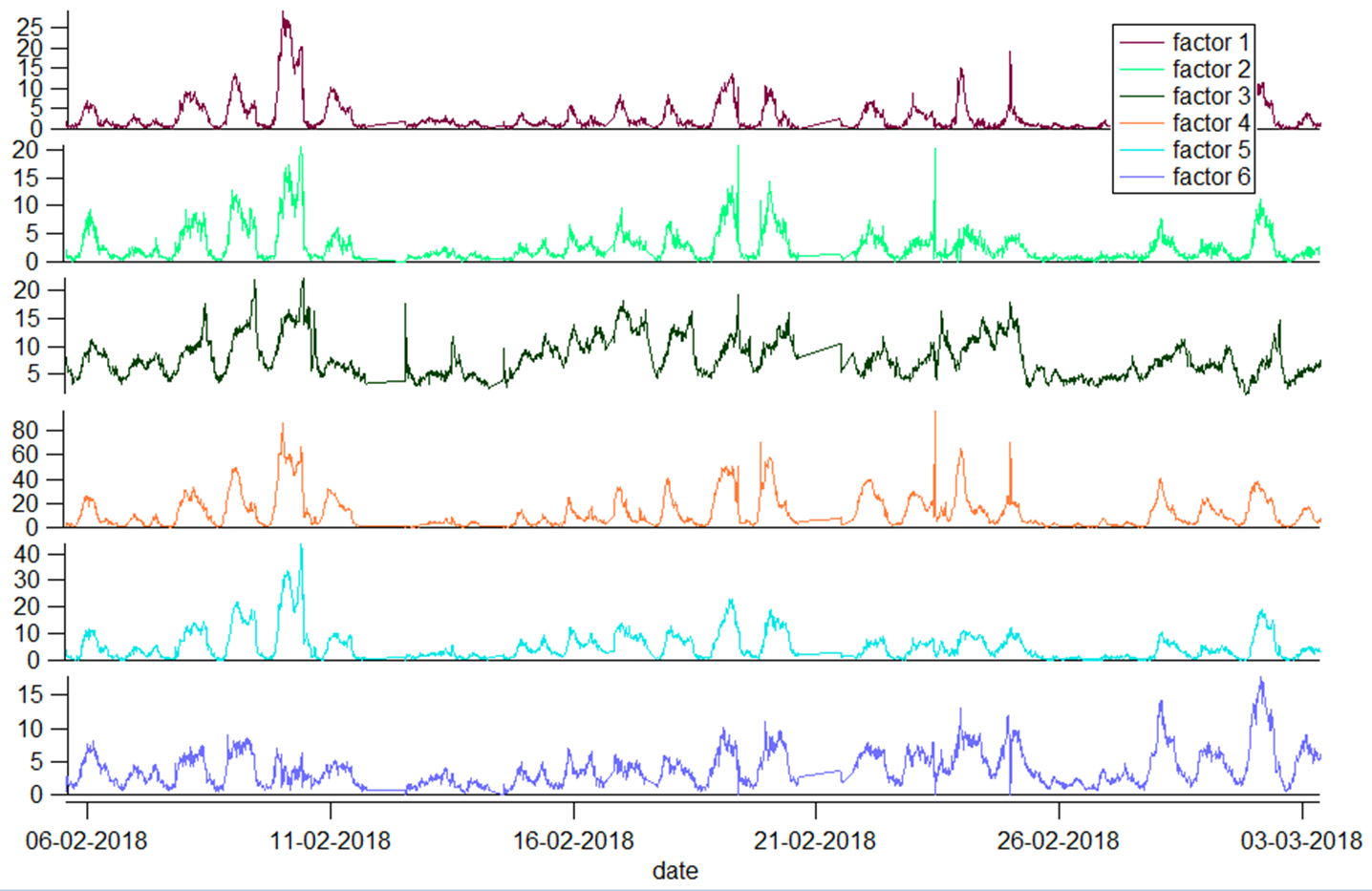

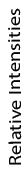

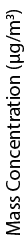


*Figure S5. Mass spectra and time series for six-factor PMF solution for seed run.*

**2. Correlation between NH_4_ predicted vs. NH_4_ measured**


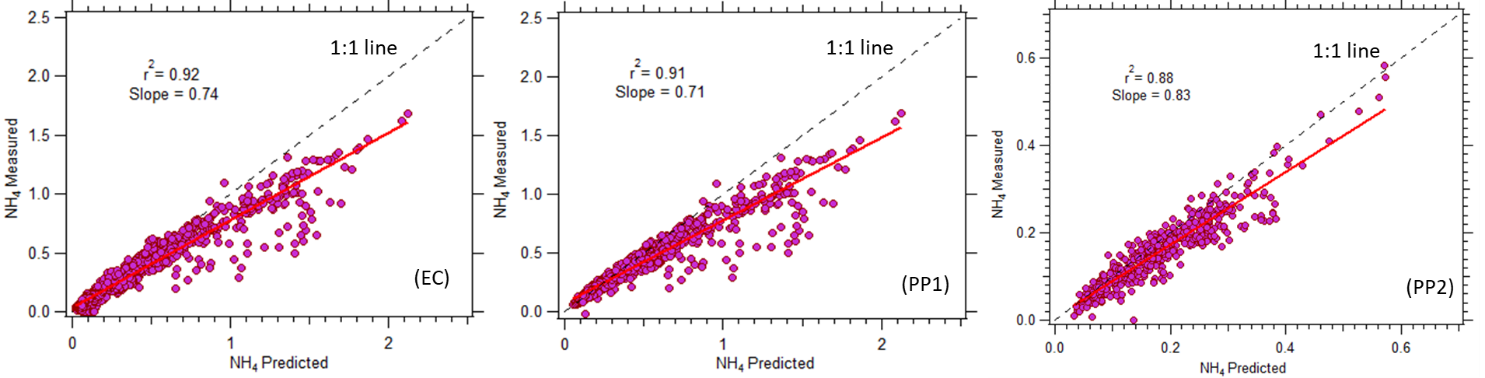


*Figure S6: Scatter plots of ammonium predicted versus ammonium measured during entire campaign (EC), pollution period 1 (PP1) with high organic high chloride concentration, pollution period 2 (PP2) with high organic low chloride concentration.*


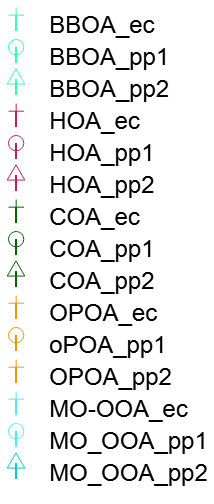

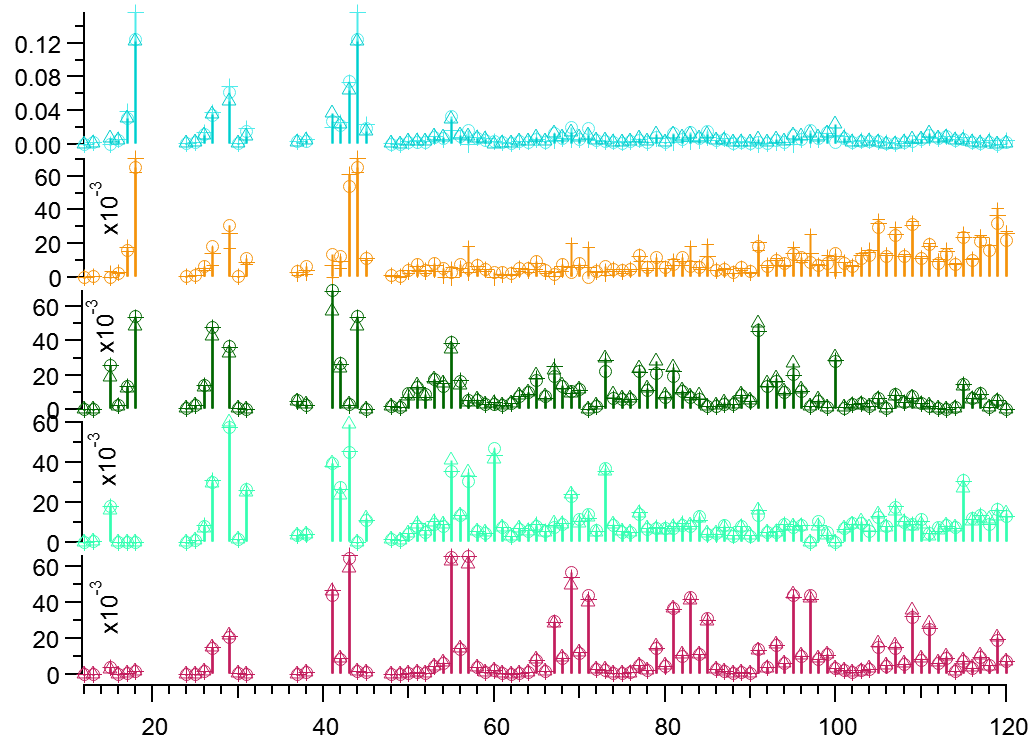

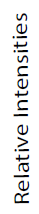

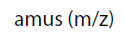


*Figure S7: Comparison between mass spectra profile of five OA components resolved from ME-2 analysis during the three separate periods; ec- entire campaign, pp1- pollution period 1, pp2- pollution period 2.*

**3. Correlation between SIA and OA components**


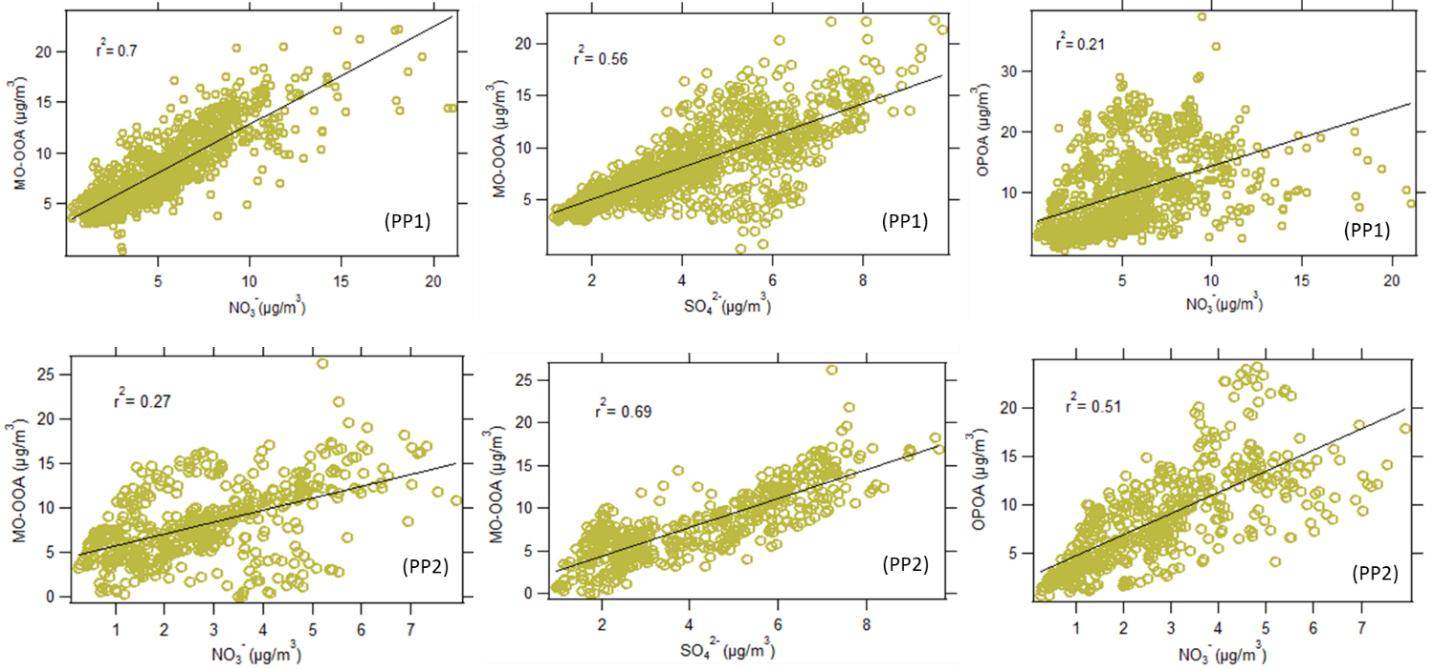


*Figure S8: Co-relation between SOA and SIA during different periods of interest.*

1. f44 vs f43


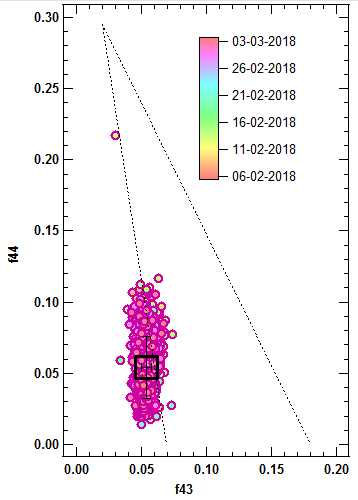

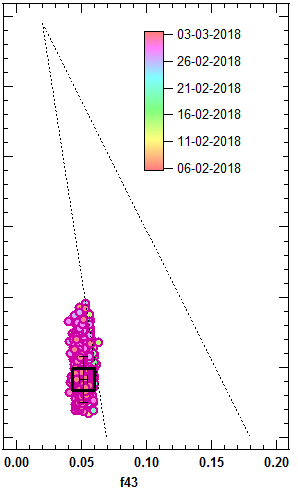

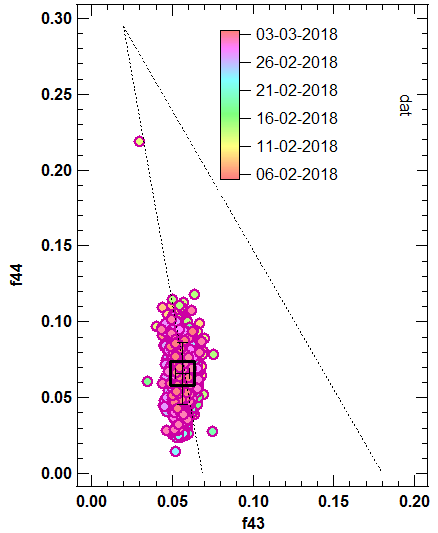


(a) Entire study

(b) Daytime

(c) Nighttime

*Figure S9. f44 vs f43 plot of OA. The triangle area is taken from Ng et al. (2010).*

**4. Selection of pollution episodes**

*Table S1. Comparison of the three selected pollution episodes. EC: Entire Campaign period; PP1: Pollution period 1; PP2: Pollution Period 2.*

|  | PP1 | PP2 | EC |
| --- | --- | --- | --- |
| Org (µg/m^3^) | 46.3 | 45.6 | 38.8 |
| Chl (µg/m^3^) | 10.1 | 1.7 | 5.9 |
| NR-PM_1_ (µg/m^3^) | 71.2 | 56.7 | 57.9 |
| O:C | 0.31 | 0.28 | 0.31 |
| H:C | 1.44 | 1.43 | 1.43 |
| HOA (%) | 41.0 | 41.2 | 46.1 |
| BBOA (%) | 12.2 | 13.4 | 13.2 |
| COA (%) | 5.1 | 7.6 | 6.7 |
| OPOA (%) | 22.8 | 19.4 | 15.4 |
| MO-OOA (%) | 19.0 | 18.3 | 18.5 |
| POA (%) | 58.3 | 62.2 | 66 |
| SOA (%) | 41.8 | 37.7 | 33.9 |

References

1. Ng, N. L. *et al.* An Aerosol Chemical Speciation Monitor (ACSM) for routine monitoring of the composition and mass concentrations of ambient aerosol. *Aerosol Sci. Technol.* **45**, 780–794 (2011).

2. Panda, U. *et al.* Evolution, composition, and chemical processes of submicron aerosols from Indian megacity: Factor analysis from Aerosol Chemical Speciation Monitor (ACSM). 10.6084/m9.figshare.27301782 (2024).
